# USP24-GSDMB complex promotes bladder cancer proliferation via activation of the STAT3 pathway

**DOI:** 10.7150/ijbs.54442

**Published:** 2021-06-11

**Authors:** Haiqing He, Lu Yi, Bin Zhang, Bin Yan, Ming Xiao, Jiannan Ren, Dong Zi, Liang Zhu, Zhaohui Zhong, Xiaokun Zhao, Xin Jin, Wei Xiong

**Affiliations:** 1 Department of Urology, The Second Xiangya Hospital, Central South University, Changsha, Hunan, 410011, China.; 2 Uro-Oncology Institute of Central South University, Changsha, Hunan, 410011, China.; 3 Cancer center, Union Hospital, Tongji Medical College, Huazhong University of Science and Technology, Wuhan, 430022, China.

**Keywords:** GSDMB, STAT3, USP24, bladder cancer

## Abstract

**Background:** Bladder cancer is the fourth and tenth most common malignancy in men and women worldwide, respectively. One of the main reasons for the unsatisfactory therapeutic control of bladder cancer is that the molecular biological mechanism of bladder cancer is complex. Gasdermin B (GSDMB) is one member of the gasdermin family and participates in the regulation of cell pyroptosis. The role of GSDMB in bladder cancer has not been studied to date.

**Methods:** TCGA database was used to exam the clinical relevance of GSDMB. Functional assays such as MTT assay, Celigo fluorescent cell-counting assay, Annexin V-APC assay and xenografts were used to evaluate the biological role of GSDMB in bladder cancer. Mass spectrometry and immunoprecipitation were used to detect the protein interaction between GSDMB and STAT3, or GSDMB and USP24. Western blot and immunohistochemistry were used to study the relationship between USP24, GSDMB and STAT3.

**Results:** In this study, bioinformatics analysis indicated that the mRNA expression level of GSDMB in bladder cancer tissues was higher than that in adjacent normal tissues. Then, we showed that GSDMB promoted bladder cancer progression. Furthermore, we demonstrated that GSDMB interacted with STAT3 to increase the phosphorylation of STAT3 and modulate the glucose metabolism and promote tumor growth in bladder cancer cells. Besides, we also showed that USP24 stabilized GSDMB to activate STAT3 signaling, which was blocked by the USP24 inhibitor.

**Conclusions:** We suggested that aberrantly up-regulated GSDMB was responsible for enhancing the growth and invasion ability of bladder cancer cells. Then, we showed that GSDMB could bind to STAT3 and activate STAT3 signaling in bladder cancer. Furthermore, we also demonstrated that USP24 interacted with GSDMB and prevented GSDMB from degradation in bladder cancer cells. Therefore, the USP24/GSDMB/STAT3 axis may be a new targetable signaling pathway for bladder cancer treatment.

## Introduction

Bladder cancer is the fourth and tenth most common malignancy in men and women worldwide, respectively 1. According to the pathological characteristics of patients with bladder cancer, clinicians choose surgical treatment, radiotherapy, chemotherapy, immunotherapy, bladder perfusion therapy, and comprehensive treatment for different stages of bladder cancer. However, each type of cancer exhibits specific problems: for non-muscle invasive bladder cancer, a high postoperative recurrence rate is the main clinical problem, while for muscle invasive bladder cancer, the prognosis is very poor, and only few patients survived more than 5 years 2, 3. One of the main reasons for the unsatisfactory therapeutic control of bladder cancer is that the molecular biological mechanism of bladder cancer is complex. Therefore, it is of great significance to identify the driving factors and inhibitors of bladder cancer and understand its mechanism of action for finding new therapeutic targets of bladder cancer.

The Janus kinases (JAK)/signal transducer and activator of transcription 3 (STAT3) axis is abnormally activated in bladder cancer and correlated with the poor prognosis 4-6. STAT3 regulates the genes associated with promoting cancer cell proliferation, invasion, and metastasis, as well as changes in metabolism 7, 8. Furthermore, STAT3 plays an important role in modulating the inflammation and immune response of the tumor microenvironment 9, 10. Thus, STAT3 is considered to be an ideal target for bladder cancer therapy. In addition to the classical IL6/JAK/STAT3 pathway 11, other pathways participate in the activation of STAT3 in bladder cancer. For instance, reactive oxygen species and glutaminolysis are reported to activate STAT3 in bladder cancer 8. Moreover, the PD-L1/ITGA6 axis promotes STAT3 nucleus translocation in bladder cancer 10. Furthermore, several proteins (e.g., EZH2 12 or Msi2 13), long non-coding RNA (e.g., SNHG16 14), and micro RNA (e.g., miR-98 14 or miR-4324) have been reported to be responsible for STAT3 activation in bladder cancer cells. Therefore, further exploring the regulatory mechanism of STAT3 in cells could provide therapeutic strategies for bladder cancer.

Gasdermin B (GSDMB) is one member of the gasdermin family and participates in the regulation of cell pyroptosis 15. The role of GSDMB in bladder cancer has not been studied to date. In this study, we systematically studied the clinical pathological and biological effects of GSDMB in bladder cancer. We found that GSDMB bound to and activated STAT3 to modulate the glucose metabolism and promote tumor growth in bladder cancer cells. Furthermore, we showed that USP24 could stabilize GSDMB, and the USP24/GSDMB/STAT3 signaling axis provided some potential therapeutic targets for bladder cancer.

## Material and Methods

### Cell lines

Bladder cancer cell lines 5637 and T24 were purchased from the Chinese Academy of Science Cell Bank (Shanghai, China). Cells were cultured in Dulbecco's modified Eagle's medium (DMEM; Thermo Fisher Scientific, China) with 10% fetal bovine serum (FBS; Thermo Fisher Scientific, China) and kept in a 37 °C incubator supplied with 5% CO_2_.

### Transfection and reagents

Cells were transfected with indicated plasmids or shRNAs using Lipofectamine 2000 (Thermo Fisher Scientific, China) according to the manufacturer's instructions. GSDMB and USP24 plasmids were purchased from Viogene Bioscience (China) and GENECHEM (China), respectively. Lentivirus-based shRNAs were obtained from Sigma-Aldrich (USA). 293T cells were transfected with shRNAs (shRNA: pVSV-G:pEQXV=1:1:2) using Lipofectamine^TM^ 2000 Transfection Reagent (Cat 11608019, Thermo Fisher Scientific, USA), culturing medium was refreshed 24 hours after transfection and then the cells were cultured for another 24 hours. After that the virus-containing medium was co-cultured with bladder cancer cell lines. 72 hours after puromycin screening, transfection-positive cells were harvested for MTS, Western blotting or xenografts analysis. Chemical reagents cryptotanshinone (Cat. No. S2285), EOAI3402143 (Cat. No. S6877), and MG132 (Cat. No. S2619) were purchased from Selleckchem.

### Immunoprecipitation and Western blot

#### Immunoprecipitation

Cells harvested from 10-cm dishes were lysed in RIPA buffer on ice for 30 min. Supernatant was collected after centrifugation (12000 g, 20 minutes) and co-culture with protein A/G beads and IgG antibody or primary antibody at 4 °C overnight. Subsequently, the beads was washed with RIPA buffer for 6 times, add 60 µL 1X loading buffer added into the beads and then boil the beads in hot water (100 °C) for 10 min.

#### Western blot

Cells harvested from 10-cm dishes were lysed in RIPA buffer on ice for 30 min. The supernatant was collected after centrifugation (12000 g, 20 minutes). Add 4X loading buffer added into the supernatant and boiled in hot water (100 °C) for 10 min. The supernatant was then subjected to electrophoresis on SDS-PAGE gels. Sealed the protein-containing PVDF membrane with 5% skim milk for 1 hour, incubate this membrane with primary antibody at 4 °C overnight. Wash the membrane with TBST buffer (3 times, 5 minutes each time) and incubate the membrane with secondary antibody for 2 hours at room temperature. Chemiluminescence Western Blot Detection Kit (Cat. No. 32209, Thermo Fisher Scientific, USA) was used to measure the chemiluminescence. Primary antibodies used for Western blotting: USP24 (Proteintech, 13126-1-AP; 1:1000 dilution), GSDMB (Proteintech, 12885-1-AP; 1:1000 dilution), GAPDH (Abcam, ab8245, 1:5000 dilution), STAT3 (Cell Signaling Technology, 30835S; 1:2000 dilution), p-STAT3 Y705 (Cell Signaling Technology, 9145S; 1:1000 dilution), and HA tag antibody (Abcam, ab18181, 1:5000 dilution). ImageJ software (National Institutes of Health) was used to evaluate protein levels.

### Glucose consumption and lactate production assay

Cells were infected with indicated constructs for 72 hours, refresh the medium and culture another 24 hours. Then the medium was collected for glucose consumption analysis and lactate production assay. Glucose (GO) assay kit (Cat. No. GAGO20, Sigma-Aldrich, USA) was used to measure glucose levels in accordance with the manufacturer's instructions. Lactic acid content determination kit (Cat. No. Use MAK064, Sigma-Aldrich, USA) was used to measure the level of lactic acid, according to the manufacturer's instructions.

### *In vitro* cell proliferation assay

#### MTT assay

Equal number of cells were placed in a 96-well plate and kept in incubator (37 °C, 5% CO2). After adding 20 µL MTT (5 mg/mL, Genview, Cat. No. JT343) reagent into each well, cells were incubated 4 hours in incubator (37 °C, 5% CO2). Remove the supernatant and add 100 µL DMSO into the supernatants and measuring the 490 nm absorbance to determine cell proliferation rate.

#### Celigo fluorescence cell-counting

Equal number of cells were placed in a 96-well plate and kept in incubator (37 °C, 5% CO2). Celigo machine (Nexcelom) was used to measure the number of cells.

### *In vivo* cell growth assay

All animal experiments were approved by the ethics committee of the Second Xiangya Hospital, Central South University. BALB/c nude mice (6 weeks old) were purchased from Vitalriver (Beijing, China). Target experiment cells (48 hours after transfection) were injected into the left back of the mice subcutaneously (2 × 10^7^ cell per mouse). Vernier caliper was used to measure the length and width of the tumors, tumor volumes were calculated using the formula (L × W^2^)/2. Once the mice were euthanized, tumors were excised and weighted.

### Quantitative real-time PCR and chromatin immunoprecipitation ChIP-qPCR

TRIzol reagent (Thermo Fisher Scientific, USA) was used to extract the total RNA from cells. cDNA was synthesized from 1 ug RNA using Reverse Transcription kit (PrimeScript™ RT reagent Kit, Code No. RR037A, Takara Bio Inc. Shigo, Japan), real-time PCR analysis was carried out with a PCR kit (TB Green™ Fast qPCR Mix, Code No. RR430A, Takara Bio Inc. Shigo, Japan) according to the manufacturer's instructions. All measured values were normalized to that of GAPDH, the 2^-ΔΔCt^ method was used to quantify fold changes. Chromatin Extraction Kit (Abcam, ab117152, USA) and ChIP Kit Magnetic - One Step (Abcam, ab156907, USA) were used to perform ChIP following the manufacturer's instructions. STAT3 (Cell signaling Technology, 30835S; 1:500) was used for the ChIP assay. Purified DNA was analyzed by real-time PCR using a PCR kit (Takara Bio Inc., Japan) according to the manufacturer's protocol.

### Tissue microarray and immunohistochemistry

Tissue microarray (Cat No. DC-Bla 1002a, Avilabio, Xian, China) was used to evaluate the relationship between USP24 and GSDMB. USP24 (Proteintech, 13126-1-AP; 1:400) and GSDMB (Proteintech, 12885-1-AP; 1:800) antibodies were used for immunohistochemistry. The immunohistochemistry score equals to the staining intensity score multiplied by the proportion of positive tumor cells. The staining intensity was graded as follows: 1= weak staining at 100× magnification but little or no staining at 40× magnification; 2 = medium staining at 40× magnification; 3 = strong staining at 40× magnification. The immunostaining intensity was evaluated for scoring independently by two experienced pathologists who were blinded for the detailed information.

### Statistical analysis

All data are presented as the mean ± standard deviation (SD). Statistical significance was determined by one- or two-way ANOVA using the GraphPad Prism 5 software. *P*-values <0.05 were considered statistically significant.

## Results

### GSDMB promotes bladder cancer progression* in vitro* and *in vivo*

**Figure 1:**
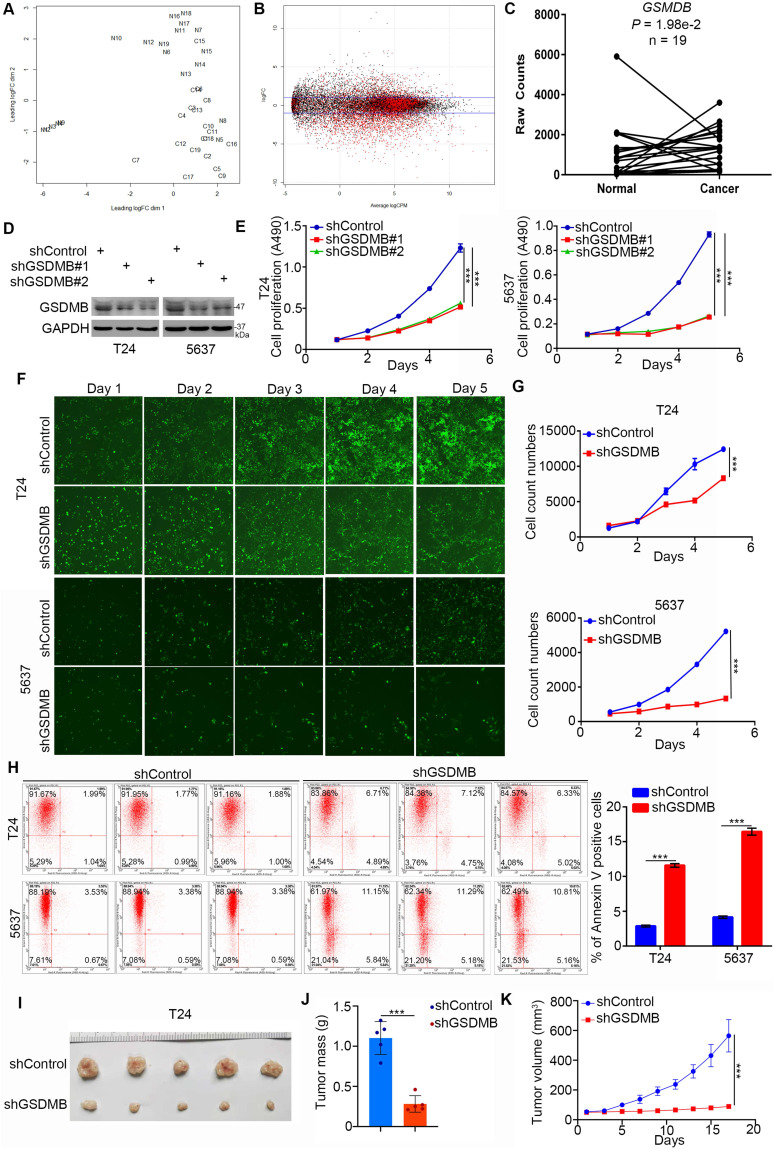
** GSDMB promotes bladder cancer progression* in vitro* and *in vivo.* A-C.** Analysis of RNA-Seq data from the TCGA dataset of bladder cancer containing 19 paired bladder cancer specimens and adjacent normal tissues. Panel A showed that normal and cancer tissues could be separated by dim2, which indicated that the data is stable for subsequent analysis. Panel B is the general linear model to estimate whether there are differences among different groups of genes. The genes with P value less than 0.05 are considered as differentially expressed genes in accordance with the zero hypothesis (the red dot in the figure). Panel C indicated that GSDMB is upregulated in the above RNA-Seq dataset with P = 1.98e-2. **D-G.** T24 and 5637 cell lines were infected with constructed plasmids. After infecting 72h, all cells were harvested for Western Blotting analysis (**D**), MTT assay (**E**) and celigo fluorescence cell count assay (**F** and** G**). All data were presented as Means ± SD (n = 3). ***, P < 0.001. **H.** T24 and 5637 cells were transfected with constructed plasmids for 72h. All cells were harvested and subjected to Annexin-V/APC assay. All data were showed as Means ± SD (n = 3). ***, P < 0.001. **I-K.** T24 cells were infected with constructed lentivirus to establish the stable knocking down cell lines. Then cells were injected subcutaneously into the nude mice to construct xenograft transplantation model. The image of xenografts was shown in (**I**), the tumor mass and volume were measured in (**J** and **K**). All data were presented as Means ± SD (n = 6). ***, P < 0.001.

First, we explored the clinical biological effects of GSDMB in bladder cancer. It is worth noting that the mRNA expression level of GSDMB in bladder cancer tissues was higher than that in adjacent normal tissues according to the bioinformatics analysis of the TCGA data set (n =19, P = 1.98e-2; Fig. 1A-C). To further study the biological role of GSDMB in bladder cancer, GSDMB was knocked down by two different short harpin RNAs (shRNAs) in both T24 and 5637 cell lines (Fig. 1D). *In vitro* cell proliferation assays showed that the growth activity of bladder cancer cells was significantly inhibited after GSDMB was knocked down (Fig. 1E-G). We also found that the apoptotic rate of bladder cancer cells in the GSDMB silencing group was significantly higher than that in the control group in both 5637 and T24 cancer cells, as analyzed by the Annexin V-APC assay (Fig. 1H). The cell cycle analysis also showed that the number of bladder cancer cells in the S phase decreased when GSDMB was knocked down (Supplementary Fig. 1A), indicating slow cell division and proliferation. In addition to the *in vitro* experiments, we also employed the nude mice xenograft tumor model after inhibition of the expression of GSDMB to study the growth-promoting effect of GSDMB in bladder cancer *in vivo*. The results revealed significantly reduced tumor volume and mass and even no tumor after knocking down GSDMB, which was consistent with the results of the *in vitro* experiments (Fig. 1I-K). Furthermore, the invasion and migration abilities of the cells were also significantly weakened after knocking down GSDMB (Supplementary Fig. 1B). Concluding, GSDMB promotes bladder cancer progression in cells and mice.

### GSDMB regulates the glycolysis of bladder cancer cells

**Figure 2:**
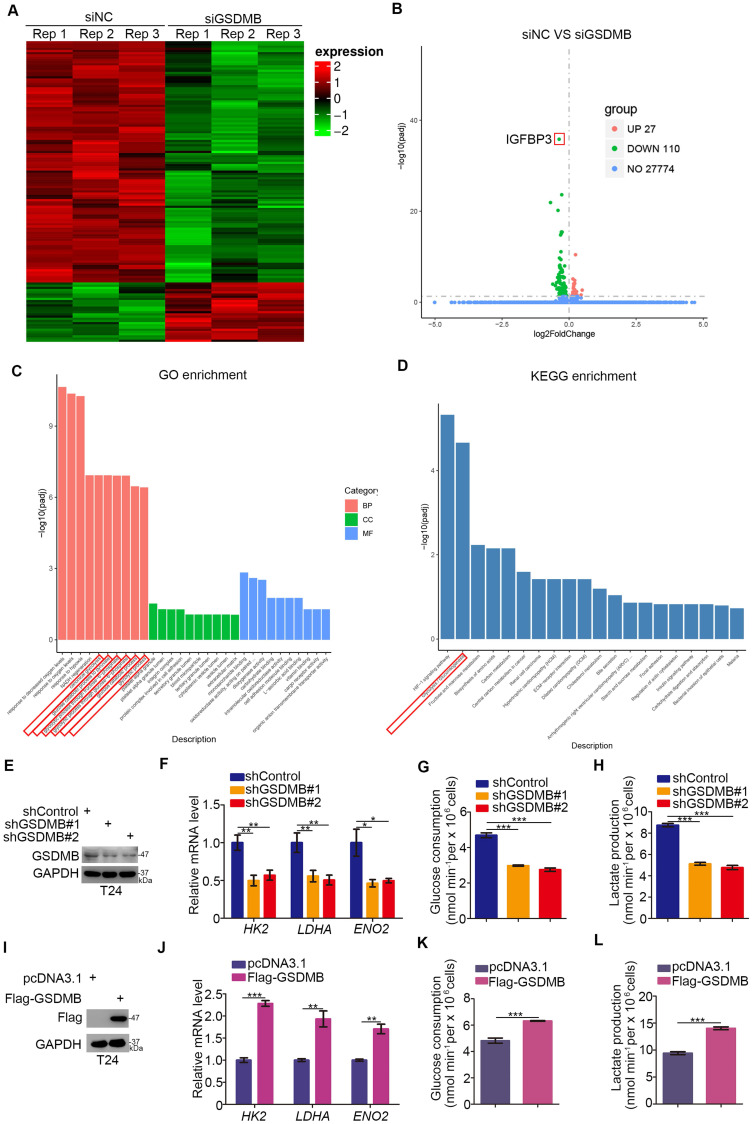
** GSDMB regulates the glycolysis of bladder cancer cells. A-B.** T24 cell lines were infected with constructed siGSDMB. After infecting 72h, cells were harvested for RNA-Seq. Heatmap (**A**) and volcano plot (**B**) were used to show the differential expressed genes. **C-D.** GO enrichment analysis (**C**) and KEGG enrichment analysis (**D**) based on differential genes through RNA-Seq. There were significant differences in glycolysis changes. **E-H.** T24 cell lines were infected with constructed plasmid. After infecting 48h and 72h, all cells were harvested for RT-qPCR (**F**) and Western Blotting analysis (**E**). Glucose consumption (original level minus remaining amount) (**G**) and lactate production (**H**) were measured in the spent medium of T24 cells. All data were presented as Means ± SD (n = 3). *, P < 0.05; **, P < 0.01; ***, P < 0.001. **I-L.** T24 cell lines were infected with constructed plasmid. After infecting 48h and 72h, all cells were harvested for RT-qPCR (**J**) and Western Blotting analysis (**I**). Glucose consumption (original level minus remaining amount) (**K**) and lactate production (**L**) were measured in the spent medium of T24 cells. All data were presented as Means ± SD (n = 3). **, P < 0.01; ***, P < 0.001.

Given the critical role of GSDMB in bladder cancer, we investigated the mechanism by which GSDMB promotes the progression of bladder cancer. We conducted RNA-Seq analysis after using small interfering RNA (siRNA) to inhibit the expression of GSDMB (Fig. 2A, B). Then, we performed GO and KEGG enrichment analysis, which indicated that GSDMB had a significant influence on glycolysis and gluconeogenesis (Fig. 2C, D). To further confirm this mechanism, we knocked down or overexpressed GSDMB in T24 cells (Fig. 2E, I, respectively). The mRNA expression levels of the genes HK2, LDHA, and ENO2, which are related to the glucose metabolism pathway, were markedly decreased when GSDMB was knocked down (Fig. 2F). Moreover, GSDMB knock down conveniently reduced glucose consumption and lactate production corresponding to the amount of GSDMB knocking down (Fig. 2G, H). In contrast, overexpression of GSDMB had the inverse effect (Fig. 2J-L). Collectively, our data suggest that GSDMB can regulate the glycolysis of bladder cancer cells.

### GSDMB regulates the glycolysis via activating STAT3 in bladder cancer cells

**Figure 3:**
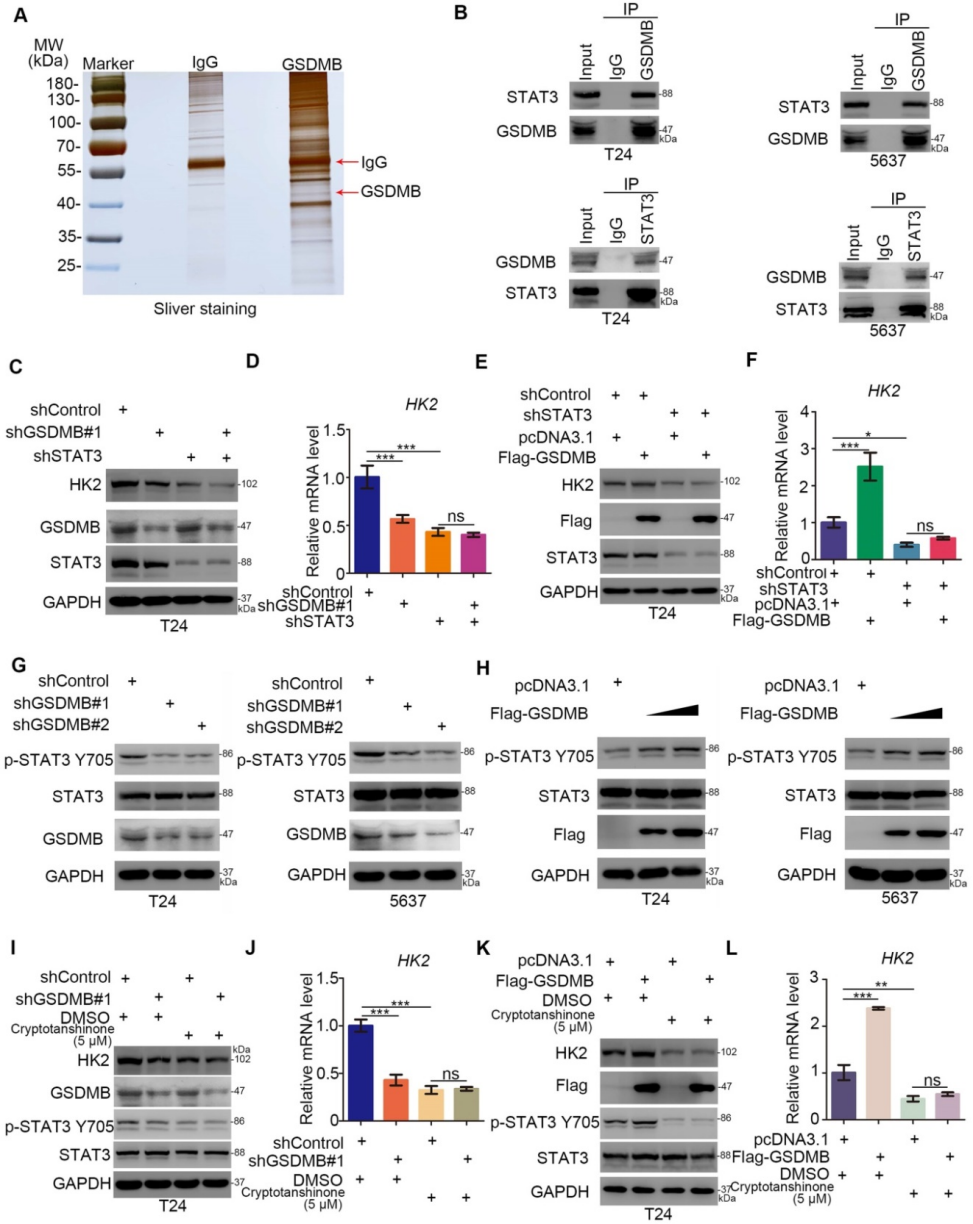
** GSDMB regulates the glycolysis via activating STAT3 in bladder cancer cells. A.** Gel electrophoresis was conducted after using GSDMB antibody for immunoprecipitation, then sliver staining was performed. The arrows indicated the protein at the expected molecular weight. **B.** Western blot analysis of co-immunoprecipitated protein samples from T24 and 5637 cells lines. **G and H.** T24 and 5637 cell lines were infected with indicated constructs (shGSDMB#1, shGSDMB#2 and Flag-GSDMB). After infecting 72h, all cells were harvested for Western Blotting analysis. **C-F.** T24 cell lines were infected with constructed plasmids (shGSDMB#1, shSTAT3 and Flag-GSDMB). After infecting 48h and 72h, all cells were harvested for RT-qPCR (**D** and **F**) and Western Blotting analysis (**C** and **D**). All data were presented as Means ± SD (n = 3). *, P < 0.05; ***, P < 0.001; ns, no significant. **I-L.** T24 cell lines were infected with constructed plasmids (shGSDMB#1 and Flag-GSDMB). After infecting 48h and 72h, the corresponding cell groups were treating with 5uM Cryptotanshinone for another 24h. Then all cells were harvested for RT-qPCR (**J** and **L**) and Western Blotting analysis (**I** and **K**). All data were presented as Means ± SD (n = 3). **, P < 0.01; ***, P < 0.001; ns, no significant.

In the following, the mechanism underlying the regulation of the glycolysis of bladder cancer cells by GSDMB was studied. We first performed mass spectrometry to identify the potential binding partners of GSDMB (Fig. 3A and Table S1), revealing that GSDMB may bind to STAT3. This finding was confirmed by immunoprecipitation in both T24 and 5637 bladder cancer cells (Fig. 3B). Multiple studies showed that STAT3 exerts a significant effect on the glycolysis and glucose-dependent addiction of tumor cells, which is known as the Warburg effect 16, 17. Specifically, it has been reported that STAT3 regulates the glucose metabolism through increasing the HK2 expression in cancer cells 18. Furthermore, STAT3 up-regulates LDHA expression to promote the proliferation of urinary bladder cancer cells 19. Interestingly, GSDMB and STAT3 proteins could precipitate each other (Fig. 3A, B). Moreover, GSDMB modulated the glycolysis by up-regulating the expression of HK2 in bladder cancer cells (Fig. 2E-2L), suggesting that GSDMB might be involved in regulating the glucose metabolism of bladder cancer cells through STAT3-related signaling pathways. To further verify that GSDMB regulated the glucose metabolism via the STAT3 pathway, we suppressed the expression of STAT3 while GSDMB was knocked down or overexpressed. The results showed that the protein and mRNA levels of HK2 remained constant after knocking down STAT3 whether GSDMB was inhibited or overexpressed (Fig. 3C-F), indicating that STAT3 is an important intermediate mediator for GSDMB in regulating the glucose metabolism. Furthermore, it has been reported that Tyr 705 STAT3 phosphorylation leads to downstream glucose metabolism disorders 20, 21. Importantly, the protein level of p-STAT3 Tyr705 was down-regulated when GSDMB was knocked down (Fig. 3G). On the contrary, p-STAT3 Tyr705 increased as the overexpression level of GSDMB increased (Fig. 3H). However, the total STAT3 level did not change whether GSDMB was knocked down or overexpressed. Furthermore, we used cryptotanshinone to inhibit the phosphorylation of Tyr 705 STAT3 in bladder cancer cells with knocking down or overexpressing GSDMB, resulting in constant protein and mRNA levels of HK2 (Fig. 3I-L). These data suggest that GSDMB regulates the glucose metabolism via promoting the phosphorylation of Tyr 705 STAT3 in bladder cancer cells.

### GSDMB-STAT3 signaling regulates IGFBP3 expression in bladder cancer

**Figure 4:**
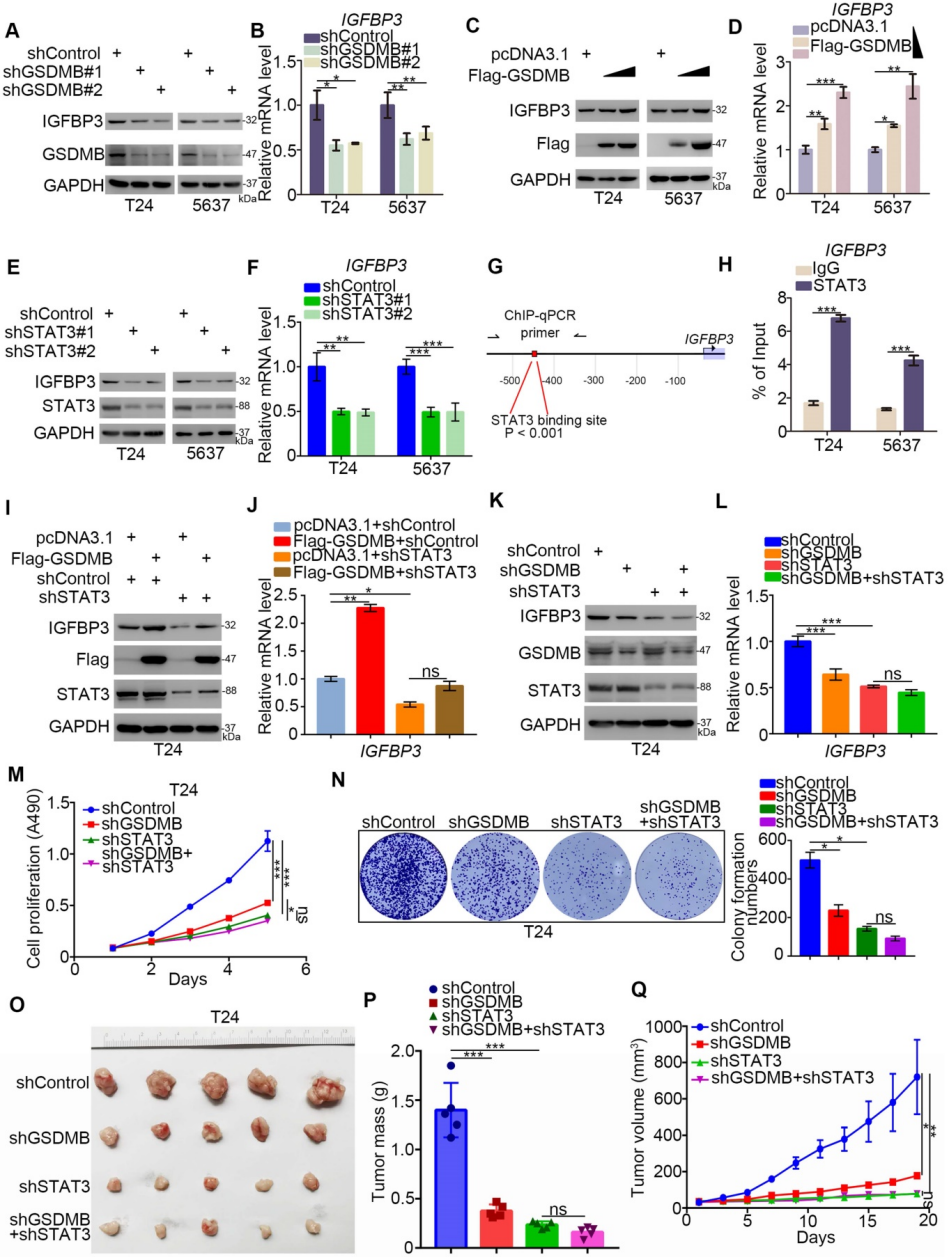
** GSDMB-STAT3 signaling regulates IGFBP3 expression in bladder cancer. A-F.** T24 and 5637 cell lines were infected with constructed plasmids (shGSDMB#1, shGSDMB#2, shSTAT3#1, shSTAT3#2 and Flag-GSDMB). After infecting 48h and 72h, all cells were harvested for RT-qPCR (**B**, **D, F**) and Western Blotting analysis (**A**, **C, E**). All data were showed as Means ± SD (n = 3). *, P < 0.05; **, P < 0.01; ***, P < 0.001. **G.** The schematic diagram of STAT3 binding to the promoter position of IGFBP3. **H.** T24 and 5637 cell lines were treated according to the protocol of ChIP experiment, and the DNA samples were performed DNA agarose gel electrophoresis. All data were showed as Means ± SD (n = 3). ***, P < 0.001. **I-L.** T24 cell lines were infected with constructed plasmids (shGSDMB, shSTAT3 and Flag-GSDMB). After infecting 48h and 72h, all cells were harvested for RT-qPCR (**J** and** L**) and Western Blotting analysis (**I** and** K**). All data were showed as Means ± SD (n = 3). *, P < 0.05; **, P < 0.01; ***, P < 0.001; ns, no significant. **M-N.** T24 cell lines were infected with constructed plasmids (shGSDMB and shSTAT3). Cells were harvested for MTS assay (**M**) and clone formation assay (**N**). All data were showed as Means ± SD (n = 3). *, P < 0.05; ***, P < 0.001; ns, no significant. **O-Q.** T24 cells were infected with constructed lentivirus to establish the stable cell lines. Then cells were injected subcutaneously into the nude mice to construct xenograft transplantation model. The image of xenografts was shown in (**O**), the tumor mass and volume were measured in (**P** and **Q**). All data were presented as Means ± SD (n = 6). ***, P < 0.001; ns, no significant.

The RNA-Seq assay revealed 27 up-regulated genes and 110 down-regulated genes with IGFBP3 as the most significantly down-regulated gene after silencing GSDMB in T24 cells. To verify the accuracy of these results, we knocked down or overexpressed GSDMB and found a positive correlation with the change in the protein and mRNA expression of IGFBP3 in both T24 and 5637 cells (Fig. 4A-D). IGFBP3 is closely related to the glucose metabolism in cells 22. The above data indicated that GSDMB may activate STAT3 to regulate the glycolysis in bladder cancer cells. Therefore, we speculated that GSDMB increased IGFBP3 in a STAT3-dependent manner. Notably, the knockdown of STAT3 inhibited the expression of IGFBP3 at the transcription and translation levels (Fig. 4E, F). Furthermore, we found that STAT3 could bind to the promoter of IGFBP3 (Fig. 4G, H), indicating that STAT3 acted as a transcription factor to initiate the transcription of IGFBP3 in bladder cancer cells. Furthermore, we knocked down the expression of STAT3 while GSDMB was knocked down or overexpressed, and the results showed that IGFBP3 remained constant (Fig. 4I-L), which confirmed our speculation. In conclusion, GSDMB regulates IGFBP3 expression through the STAT3 pathway in bladder cancer.

Moreover, the rate of cell proliferation did not significantly change after the simultaneous knocking down of GSDMB and STAT3, while it decreased after STAT3 was knocked down alone (Fig. 4M, N). The results of the *in vivo* experiments were also completely consistent with these results, and no statistical difference in the tumor mass or volume was detected between the two groups, which further demonstrated that GSDMB promoted the progression of bladder cancer by regulating STAT3.

### USP24 interacts with GSDMB to up-regulate the GSDMB protein level in bladder cancer

**Figure 5:**
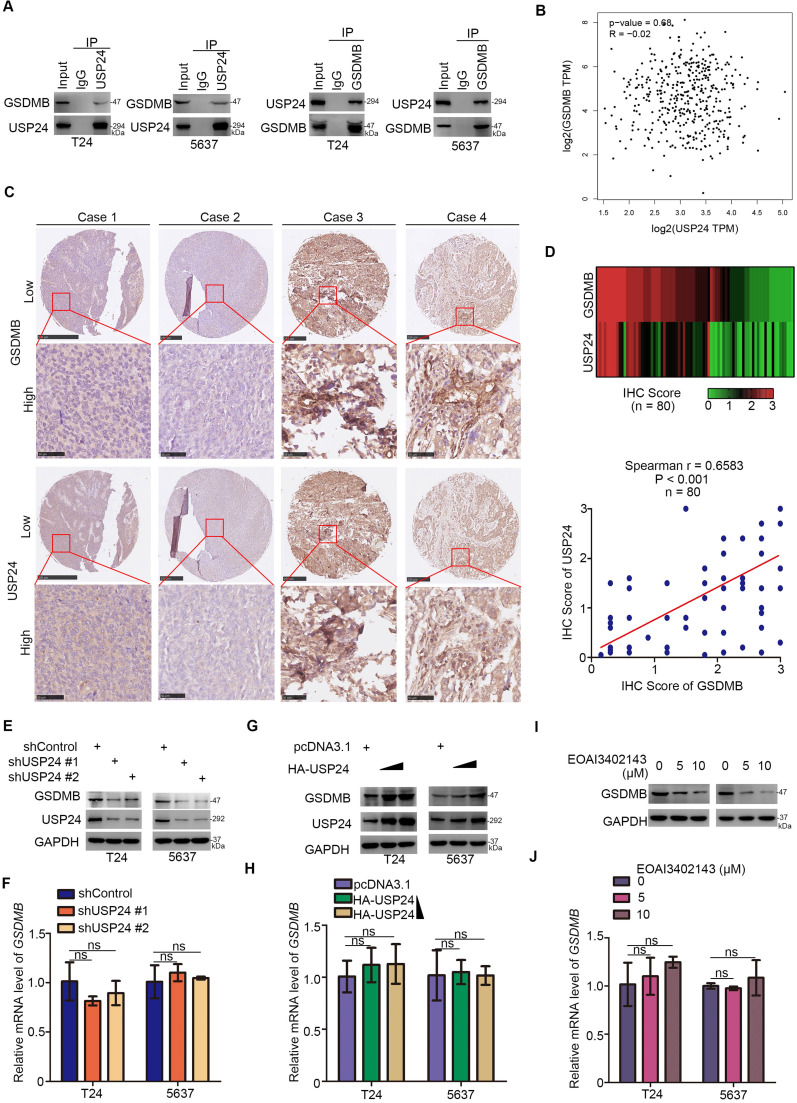
** USP24 interacts with GSDMB to up-regulate the GSDMB protein level in bladder cancer. A.** Western blot analysis of co-immunoprecipitated protein samples from T24 and 5637 cells lines. **B.** GEPIA network tool analyzed the RNA correlation between GSDMB and USP24. **C.** The typical IHC staining images using TMA tissue chip (n = 80). **D**. Heatmap and scatter diagram were used to reveal the correlation of protein expression between GSDMB and USP24 in bladder cancer patient specimens. (n=80, spearman correlation r = 0.6583, P < 0.001). **E-H.** T24 and 5637 cell lines were infected with constructed plasmids (shUSP24#1, shUSP24#2, HA-USP24). After infecting 48h and 72h, all cells were harvested for RT-qPCR (**F** and **H**) and Western Blotting analysis (**E** and **G**). All data were showed as Means ± SD (n = 3). ns, no significant. **I-J.** T24 and 5637 cell lines were treated with EOAI3402143 for 48h and 72h. Then cells were harvested for RT-qPCR (**I**) and Western Blotting analysis (**J**). All data were showed as Means ± SD (n = 3). ns, no significant.

After revealing the important role of GSDMB in bladder cancer, we studied how GSDMB was regulated in bladder cancer for the targeted treatment of bladder cancer. Mass spectrometry of GSDMB indicated that GSDMB may interact with USP24 (Table S1), which was verified in bladder cancer cells through immunoprecipitation (Fig. 5A). Interestingly, GEPIA network tool analysis showed that there was no correlation between GSDMB and USP24 in their RNA levels (Fig. 5B). To analyze the protein expression levels of GSDMB and USP24, a bladder cancer tissue microarray (n = 80) was performed and obtained typical images are displayed in Fig. 5C. The results showed a significant positive correlation between GSDMB and USP24 proteins in clinical specimens (Fig. 5D). As a deubiquitinating enzyme, ubiquitin-specific peptidase USP24 could deubiquitinate downstream proteins to maintain their stability 23. Thus, USP24 may mediate the deubiquitination of GSDMB and maintain its stability. Consistently, the protein expression level of GSDMB also decreased or increased after knockdown or overexpression of USP24, respectively, while the mRNA level of GSDMB remained constant (Fig. 5E-H). Furthermore, the changes in GSDMB observed after treatment with USP24 inhibitor EOAI3402143 were exactly the same as those observed after knocking down USP24 (Fig. 5I, J). To sum up, USP24 interacts with GSDMB to up-regulate the GSDMB protein level in bladder cancer.

### USP24 stabilizes GSDMB to promote STAT3 phosphorylation in bladder cancer cells

**Figure 6:**
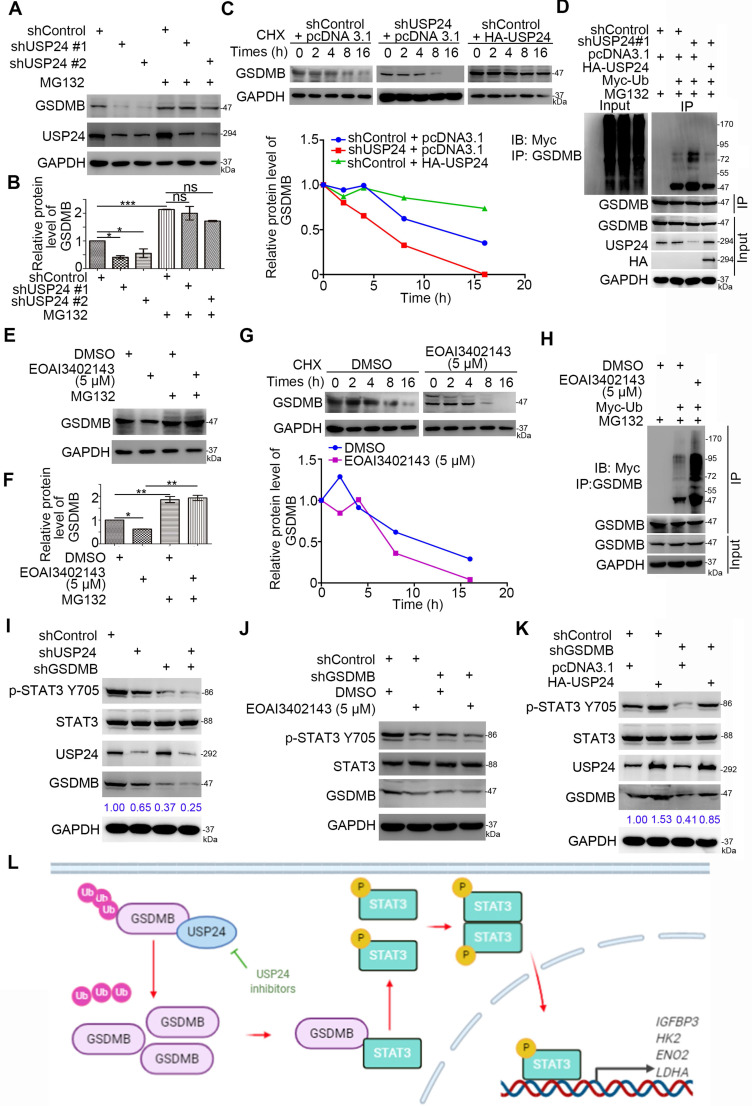
**USP24 stabilizes GSDMB to promote STAT3 phosphorylation in bladder cancer cells. A-B.** T24 and 5637 cell lines were infected with constructed plasmids (shUSP24#1, shUSP24#2). After infecting 48h and 72h, the corresponding groups were treated with MG132 for another 12h. All cells were harvested for Western Blotting analysis.** C.** T24 cells were infected with indicated plasmids (shUSP24 and HA-USP24). After infecting 72h, cells were treated with Cycloheximide (CHX) and all cells were collected for Western Blotting analysis at different time points. **D.** T24 cells were infected with indicated plasmids (shUSP24#1, HA-USP24 and Myc-Ub). After 24h, cells were treated with MG132 for 12h. Then all cells were collected for Western Blotting analysis. **E-F.** T24 cells were treated with 5uM EOAI3402143. After 48h, cells were treated with MG132 for another 24h. Then all cells were collected for Western Blotting analysis. **G.** T24 cells were treated with 5uM EOAI3402143. After 72h, cells were treated with Cycloheximide (CHX) and all cells were collected for Western Blotting analysis at different time points. **H.** T24 cells were treated with 5uM EOAI3402143. After 48h, T24 cells were infected with indicated plasmids (Myc-Ub) for 24h. Then cells were treated with MG132 for 12h. All cells were collected for Western Blotting analysis. **I-K.** T24 cell lines were infected with constructed plasmids (shUSP24, shGSDMB, HA-USP24). Cells were treated with 5uM EOAI3402143 in (**J**). After infecting or treating 72h, all cells were harvested for Western Blotting analysis. **L.** The schematic diagram for USP24 stabilizing GSDMB to promote STAT3 phosphorylation, which subsequently increased the expression of glucose metabolism-related genes (e.g., *HK2, LDHA, ENO2, and IGFBP3*) and promoted bladder cancer cell proliferation.

Given that USP24 promoted the expression of the GSDMB protein levels rather than that of the mRNA levels in bladder cancer cells, we hypothesized that USP24 may regulate the stability of GSDMB through the ubiquitinated proteasome pathway. We have shown that knocking down USP24 using shUSP24 or USP24 inhibitor EOAI3402143 could reduce the protein expression of GSDMB, but the process was inhibited by proteasome inhibitor MG132 (Fig. 6A, B, E, F). Moreover, the protein half-life of GSDMB was significantly decreased when USP24 was knocked out or inhibited, while overexpression of USP24 showed the opposite effect (Fig. 6C, G). The proteasome recognized and degraded ubiquitinated proteins 24. We proved that knocking down or inhibiting USP24 increased the polyubiquitination of GSDMB, but overexpressing USP24 decreased the polyubiquitination of GSDMB in bladder cancer cells (Fig. 6D, H). These data suggest that USP24 promoted the stability of the GSDMB protein in bladder cancer by deubiquitinating GSDMB.

Moreover, after USP24 was inhibited or overexpressed, the expression level of p-STAT3 Y705 also decreased or increased, respectively (Fig. 6I-K). This raised the question of whether USP24 regulated the activation of the STAT3 signaling pathway through GSDMB. To verify this hypothesis, we knocked down the expression of GSDMB while knocking down or suppressing the expression of USP24. The results showed that the p-STAT3 Y705 protein level remained stable, while it decreased when GSDMB was knocked down alone (Fig. 6I, J). Similarly, overexpressing USP24 after knocking down GSDMB also resulted in a constant p-STAT3 Y705 protein level (Fig. 6K). Therefore, the USP24-GSDMB pathway axis played a key role in modulating the phosphorylation of STAT3. In conclusion, USP24 stabilizes GSDMB to promote STAT3 phosphorylation in bladder cancer cells (Fig. 6L).

## Discussion

Gasdermin (GSDM) was first found in the epithelial tissues of the gastrointestinal tract and skin and consists of the six members GSDMA, GSDMB, GSDMC, GSDMD, GSDME, and DFNB59 25, 26. GSDM gene family members have about 45% sequence homology and two domains that can bind to each other 26. The N-terminal domain can oligomerize in the cell membrane and form a pyrolytic hole with 10-16 nm in diameter 27. Smaller intracellular substances, such as interleukin (IL)-1β and IL-18, can be secreted through this hole. Increased pyrolytic pores eventually lead to cell scorch and release the whole cell substance 27. As one member of the GSDM family, GSDMB is cleaved by caspase-1, and the release of the N-terminal domain induces cell death 15. So far, the role of GSDMB in tumors is still controversial. Some studies showed that GSDMB can promote pyroptotic death and inhibit the growth of tumor cells 15. In contrast, in HER2-positive breast cancer patients, the clinical outcome of patients with high expression of GSDMB (about 60% of patients) is significantly worse, and the neoadjuvant effect is poor, which is often accompanied by trastuzumab resistance 28, 29. Delivery of nano antibodies against GADMB has shown an obvious inhibitory effect on breast cancer *in vivo* and *in vitro* levels 30. At present, the specific role and underlying mechanism of GSDMB in bladder cancer need to be further explored. In this study, we showed that GSDMB was up-regulated in bladder cancer tissues compared with adjacent normal tissue. Overexpressed GSDMB promoted cancer cell growth via interacting with STAT3 to elevate the phosphorylation level of STAT3, which increased the expression of *HK2, LDNA, ENO2,* and *IGFBP3* to enhance the glycolysis of bladder cancer cells. However, the mechanism underlying the modulation of the phosphorylation of STAT3 by GSDMB is still not fully understood, so further experimental studies are needed.

Ubiquitin-specific peptidase 24 (USP24), containing 2620 amino acids, acted as a deubiquitinase to regulate the ubiquitin modification of the substrates 31. The biological role of USP24 in cancer is poorly understood. It has been reported that USP24 is overexpressed in the late stage of lung cancer 32. Up-regulated USP24 increased the protein level of β-TrCP and p300 to promote lung cancer malignancy 31. Moreover, targeting USP24 by small inhibitors hinders the progression of T-cell acute lymphoblastic leukemia and B-cell malignancies 33, 34. However, some groups also demonstrated that USP24 prevents P53 from degradation to regulate the DNA damage response 23. Intriguingly, USP24 up-regulates the expression of MDM2, the well-known E3 ligase of P53, in lung cancer, which contradicts the above finding 31. Thus, the role of USP24 in cancer needs to be further elucidated. Here, our results indicated that USP24 bound to GSDMB to stabilize GSDMB, and subsequently activated the STAT3 pathway in bladder cancer cells. We further showed that USP24 inhibitors could block this process via inducing GSDMB degradation in cancer cells, which provided a therapeutic strategy for inhibiting the GSDMB/STAT3 axis in bladder cancer. In contrast, it has been reported that EOAI3402143, which we used as a USP24 inhibitor, not only inhibits the activity of USP24 but also targets USP9x and USP5 in cells 34. Although we genetically silenced USP24 to prove the stabilization of GSDMB by USP24, we could not rule out that USP9x or USP5 acted as deubiquitinases of GSDMB in bladder cancer.

In summary, we demonstrated that aberrantly up-regulated GSDMB was responsible for enhancing the growth and invasion ability of bladder cancer cells. Then, we showed that GSDMB could bind to STAT3 and elevate the phosphorylation of STAT3, which may be the mechanism underlying the transcriptional increase of the expression of glucose metabolism-related genes (e.g., *HK2, LDHA, ENO2, and IGFBP3*) and the promotion of cancer cell proliferation in bladder cancer by GSDMB. Furthermore, we also demonstrated that USP24 interacted with GSDMB and prevented GSDMB from degradation in bladder cancer cells. Therefore, the USP24/GSDMB/STAT3 axis may be a new targetable signaling pathway for bladder cancer treatment.

## Supplementary Material

Supplementary figure and tables.

## Abbreviations

GSDMB: Gasdermin B
STAT3: signal transducer and activator of transcription 3
JAK: Janus kinases
USP24: Ubiquitin-specific peptidase 24
ChIP: Chromatin immunoprecipitation
IHC: immunohistochemistry
MTT: 3-(45)-dimethylthiahiazo (-z-y1)-35-di-phenytetrazoliumromide
