# Supplementary material for: USP24-GSDMB complex promotes bladder cancer proliferation via activation of the STAT3 pathway
Source: Int J Biol Sci. 2021 Jun 11;17(10):2417–29. doi: 10.7150/ijbs.54442 (PMC8315027; doi:10.7150/ijbs.54442)
Supplement: Supplementary file 1 — Supplementary figure and tables. [file ijbsv17p2417s1.pdf]

USP24-GSDMB complex promotes bladder cancer proliferation via activation of the STAT3 pathway

Haiqing He, Lu Yi, Bin Zhang, Bin Yan, Ming Xiao, Jiannan Ren, Dong Zi, Liang Zhu, Zhaohui Zhong, Xiaokun Zhao, Xin Jin, Wei Xiong

Supplementary figure 1

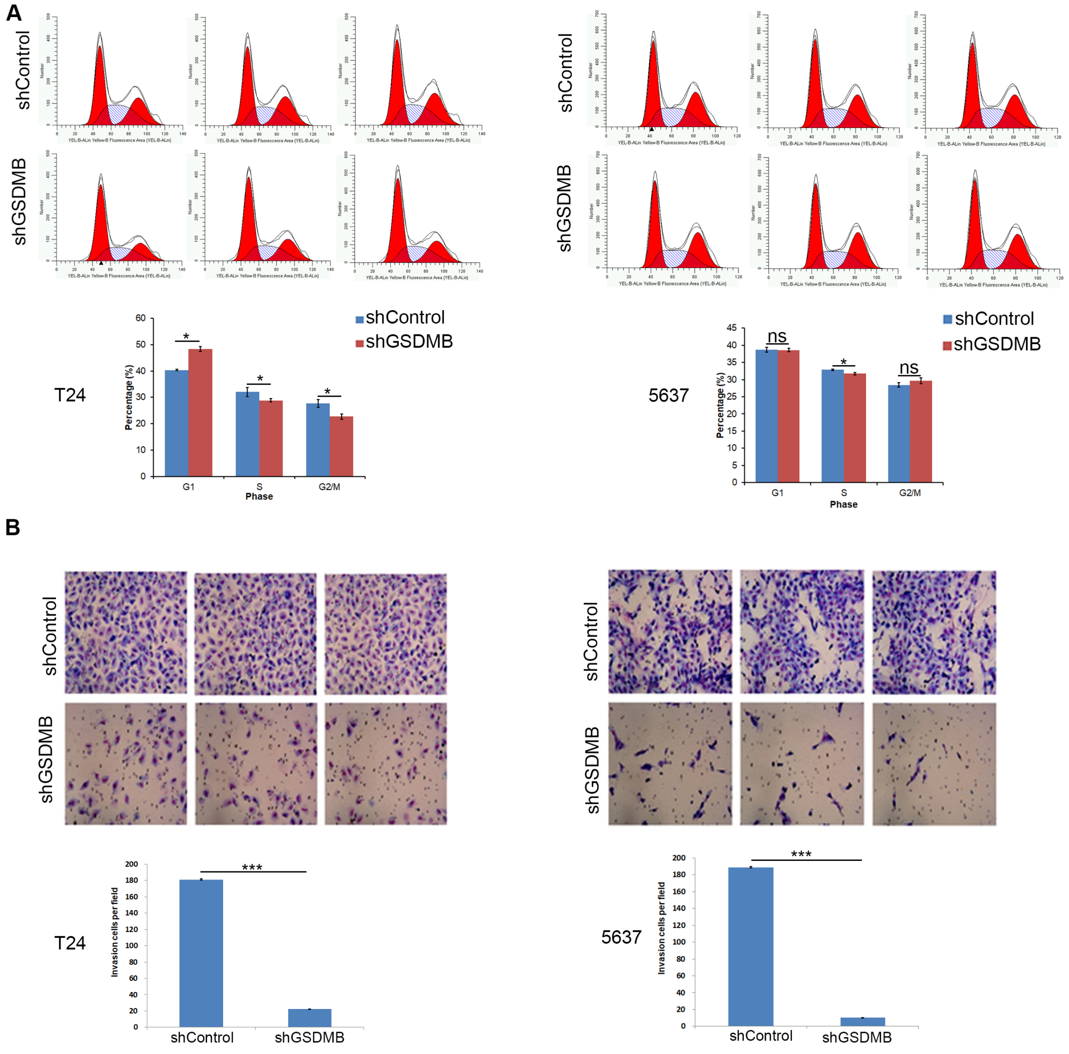

Supplementary figure 1. A and B, T24 and 5637 cells were infected with indicated constructs for 48 h. The cells were harvested for cell cycle assay (A) and transwell invasion assay (B). Data

presented as three replicates. ns, not significant; *, $P < 0.05$; ***, $P < 0.001$.

Table S1: Sequences of RT-qPCR primers

Species	Gene	Forward (5'-3')	Reverse (5'-3')
Human	*GAPDH*	CCAGAACATCATCCCTGCCT	CCTGCTTCACCACCTTCTTG
Human	*HK2*	TCTATGCCATCCCTGAGGAC	TCTCTGCCTTCCACTCCACT
Human	*LDHA*	GGCCTGTGCCATCAGTATCT	CGCTTCCAATAACACGGTTT
Human	*ENO2*	GAAGAAAAGGCCTGCAACTG	CCAGGTCAGCAATGAATGTG
Human	*GSDMB*	AGTCTTTGGGTTCGGAGGAT	CTTGTCTGGGTCCTCCATGT
Human	*IGFBP3*	CCTGCCGTAGAGAAATGGAA	AGGCTGCCCATACTTATCCA

Table S2: Sequences of ChIP-qPCR primers

Species	Gene	Forward (5'-3')	Reverse (5'-3')
Human	*IGFBP3*	CCCCTGGGGATATAAACAGC	GCATTCGTGTGTACCTCGTG

Table S3: Sequences of gene-specific shRNAs

shGSDMB-1	5′-CCGGGCCTTGTTGATGCTGATAGATCTCGAGATCTATCAGCATCAACAAGGCTTTT[illegible]G-3′
shGSDMB-2	5′-CCGGGCTGTATGTTGTTGTCTCTATCTCGAGATAGAGACAACAACATACAGCTTTTTTG-3′

shSTAT3	5′-<br>CCGGGCACAATCTACGAAGAATCAACTCGAGTTGATTCTTCGTAGATTGTGCTTTT[illegible]<br>-3′
shUSP24-1	5′-<br>CCGGCTCTCGTATGTAACGTATTTGCTCGAGCAAATACGTTACATACGAGAGTTTT[illegible]<br>TG-3′
shUSP24-2	5′-<br>CCGGACAATACTGTGACCGTATAAACTCGAGTTTATACGGTCACAGTATTGTTTTT[illegible]<br>-3′

gene_id	si-GSDMB	si-NC	log2Fold(	pvalue	padj	gene_nam(	gene_chr	gene_sta(
ENSG0000(	10533.42	13568.93	-0.36519	3.59E-40	1.51E-36	IGFBP3	7	45912245
ENSG0000(	19424.15	23403.74	-0.26886	8.19E-28	2.30E-24	GPRC5A	12	12890782
ENSG0000(	794.1105	1283.043	-0.69282	6.31E-26	1.33E-22	ANGPTL4	19	8363289
ENSG0000(	2922.117	3881.46	-0.40926	3.51E-24	5.92E-21	ERRFI1	1	8004404
ENSG0000(	9568.855	11410.67	-0.25407	2.81E-19	3.65E-16	ITGA5	12	54395261
ENSG0000(	7126.705	8668.058	-0.28243	3.03E-19	3.65E-16	SERPINE1	7	1.01E+08
ENSG0000(	5856.391	7176.623	-0.29352	7.35E-19	7.74E-16	SLC6A6	3	14402576
ENSG0000(	5884.25	7292.295	-0.30982	1.83E-18	1.71E-15	HK2	2	74833981
ENSG0000(	6360.943	7854.364	-0.30454	8.61E-15	7.26E-12	SLC6A8	X	1.54E+08
ENSG0000(	7191.42	6072.767	0.244017	4.92E-14	3.77E-11	TFRC	3	1.96E+08
ENSG0000(	2038.861	2618.166	-0.36075	2.70E-13	1.90E-10	FN1	2	2.15E+08
ENSG0000(	2907.643	3586.16	-0.303	6.19E-13	4.01E-10	NOTCH3	19	15159038
ENSG0000(	2332.8	2949.548	-0.33877	1.08E-11	6.48E-09	ADM	11	10304680
ENSG0000(	1696.743	2164.6	-0.35154	1.69E-11	9.49E-09	ALDOC	17	28573115
ENSG0000(	23307.92	26182.69	-0.16782	1.86E-11	9.82E-09	PGK1	X	77910739
ENSG0000(	1907.913	2376.686	-0.31705	4.94E-11	2.44E-08	FAM162A	3	1.22E+08
ENSG0000(	1716.833	2165.356	-0.33526	5.22E-11	2.44E-08	ENO2	12	6913745
ENSG0000(	2577.481	3204.537	-0.31354	6.01E-11	2.67E-08	BNIP3	10	1.32E+08
ENSG0000(	1843.652	2345.482	-0.34672	6.78E-11	2.86E-08	MXI1	10	1.1E+08
ENSG0000(	2496.07	3073.701	-0.29996	1.26E-10	5.07E-08	BNIP3L	8	26382898
ENSG0000(	2756.139	3294.467	-0.25724	2.16E-10	8.26E-08	P4HA1	10	73007217
ENSG0000(	4715.613	5587.321	-0.2449	6.19E-10	2.27E-07	TTYH3	7	2631951
ENSG0000(	4154.922	4980.215	-0.26172	6.59E-10	2.32E-07	STC2	5	1.73E+08
ENSG0000(	1523.892	1912.704	-0.32812	6.90E-10	2.33E-07	PFKFB4	3	48517684
ENSG0000(	969.432	1263.778	-0.38245	2.35E-09	7.61E-07	PLIN2	9	19108375
ENSG0000(	1479.844	1866.864	-0.33592	6.10E-09	1.90E-06	SH3BP2	4	2793023
ENSG0000(	1180.706	1513.73	-0.35855	7.09E-09	2.13E-06	ITGA2	5	52989326
ENSG0000(	4942.48	5751.884	-0.21911	8.13E-09	2.24E-06	SLC2A1	1	42925375
ENSG0000(	3668.598	4338.151	-0.24138	8.15E-09	2.24E-06	PLOD2	3	1.46E+08
ENSG0000(	2162.38	2649.358	-0.29357	8.25E-09	2.24E-06	SEMA4B	15	90160604
ENSG0000(	5840.27	6622.783	-0.18122	1.31E-08	3.45E-06	ASPH	8	61500556
ENSG0000(	651.8366	875.5015	-0.42633	1.44E-08	3.67E-06	EGLN3	14	33924231
ENSG0000(	15407.55	17695.16	-0.19958	1.68E-08	4.07E-06	MT-RNR1	MT	648
ENSG0000(	1190.78	1509.866	-0.34164	1.69E-08	4.07E-06	LOX	5	1.22E+08
ENSG0000(	15359.87	13834.96	0.15075	3.40E-08	7.95E-06	CLPTM1L	5	1317744
ENSG0000(	704.5072	924.0164	-0.39067	6.74E-08	1.53E-05	NXPH4	12	57216795
ENSG0000(	4364.424	3774.178	0.209326	7.89E-08	1.75E-05	CYR61	1	85580761
ENSG0000(	913.6418	1160.109	-0.34433	8.24E-08	1.78E-05	FAM13A	4	88725955
ENSG0000(	840.5915	1093.296	-0.3799	1.49E-07	3.13E-05	C1QL1	17	44959693
ENSG0000(	537.6951	741.7121	-0.46481	2.33E-07	4.80E-05	MIR210HG	11	565660
ENSG0000(	5127.601	5853.988	-0.19075	3.16E-07	6.34E-05	ANLN	7	36389806
ENSG0000(	1034.337	1300.021	-0.32928	3.44E-07	6.75E-05	LUCAT1	5	91054834
ENSG0000(	4752.305	4105.317	0.210789	3.62E-07	6.93E-05	ID3	1	23557918
ENSG0000(	1464.366	1801.887	-0.30001	4.72E-07	8.84E-05	KDM4B	19	4969113
ENSG0000(	5875.843	5199.976	0.176194	4.84E-07	8.87E-05	CTPS1	1	40979335
ENSG0000(	1773.929	2155.081	-0.28143	5.26E-07	9.44E-05	ITGB4	17	75721328
ENSG0000(	1343.755	1639.256	-0.28647	5.90E-07	0.000104	FUT11	10	73772291
ENSG0000(	217.1118	332.0756	-0.61343	6.55E-07	0.000113	CDCP1	3	45082278
ENSG0000(	5196.037	5904.274	-0.18467	7.27E-07	0.000123	LOXL2	8	23297189
ENSG0000(	6743.989	5923.751	0.187162	8.26E-07	0.000136	TM4SF1	3	1.49E+08
ENSG0000(	419.8122	597.7657	-0.50852	1.35E-06	0.000216	PDK1	2	1.73E+08

ENSG0000(	5539.729	6178.067	-0.15752	1.36E-06	0.000216	BHLHE40	3	4979116
ENSG0000(	16494.27	18127.07	-0.13625	2.17E-06	0.000337	TPI1	12	6867119
ENSG0000(	2390.554	2766.624	-0.21107	2.20E-06	0.000337	TGFB1	19	41301587
ENSG0000(	5859.12	5216.463	0.16778	2.26E-06	0.00034	FSTL1	3	1.2E+08
ENSG0000(	46097.42	49941.95	-0.11552	4.23E-06	0.000625	LDHA	11	18394388
ENSG0000(	1262.941	1522.619	-0.26999	4.46E-06	0.000648	C11orf86	11	66975277
ENSG0000(	562.2665	742.432	-0.40088	4.62E-06	0.00066	HILPDA	7	1.28E+08
ENSG0000(	617.2174	791.4312	-0.35852	4.97E-06	0.000698	EDN2	1	41478775
ENSG0000(	4986.492	4457.594	0.161637	5.75E-06	0.000795	PRSS23	11	86791059
ENSG0000(	6942.402	7697.18	-0.14866	6.20E-06	0.000843	ATP1B1	1	1.69E+08
ENSG0000(	1625.468	1905.602	-0.22902	8.47E-06	0.001134	WSB1	17	27294076
ENSG0000(	369.8956	506.0436	-0.45142	9.70E-06	0.001278	TMEM158	3	45224466
ENSG0000(	8215.586	9367.34	-0.18954	1.07E-05	0.001392	DDIT4	10	72273920
ENSG0000(	786.6786	988.6582	-0.32867	1.34E-05	0.001685	ARRDC3	5	91368724
ENSG0000(	1620.694	1904.193	-0.23268	1.34E-05	0.001685	KDM3A	2	86440647
ENSG0000(	872.9347	1079.129	-0.30565	1.37E-05	0.0017	AP000769.	11	65455258
ENSG0000(	3257.073	2816.573	0.209878	1.46E-05	0.001785	OLR1	12	10158301
ENSG0000(	2046.425	2351.848	-0.20049	1.57E-05	0.001884	FAM107B	10	14518557
ENSG0000(	537.2425	695.5082	-0.37359	1.59E-05	0.001884	NDUFA4L2	12	57234903
ENSG0000(	399.2755	285.5651	0.484539	1.77E-05	0.002071	EEA1	12	92770637
ENSG0000(	2071.52	2417.581	-0.22355	1.92E-05	0.002221	H1F0	22	37805093
ENSG0000(	2534.479	2902.948	-0.1964	1.95E-05	0.002225	QSOX1	1	1.8E+08
ENSG0000(	1813.703	2104.11	-0.21431	2.37E-05	0.002666	SLC16A6	17	68267026
ENSG0000(	1251.651	1484.11	-0.24577	2.80E-05	0.003106	SFMBT2	10	7158624
ENSG0000(	682.1704	860.1644	-0.33498	3.12E-05	0.003421	PPFIA4	1	2.03E+08
ENSG0000(	1213.279	1435.934	-0.24278	3.64E-05	0.003891	BACH1	21	29194071
ENSG0000(	8004.565	8780.411	-0.13329	3.65E-05	0.003891	BZW1	2	2.01E+08
ENSG0000(	1931.257	2218.608	-0.20017	4.10E-05	0.004318	EFNA1	1	1.55E+08
ENSG0000(	3032.078	2670.233	0.183725	4.34E-05	0.004511	DAB2	5	39371675
ENSG0000(	14682.12	15921.71	-0.11702	4.39E-05	0.004511	GPI	19	34359480
ENSG0000(	1127.596	927.8047	0.280557	4.86E-05	0.00494	HSPA1A	6	31815464
ENSG0000(	7077.938	6437.234	0.136735	5.20E-05	0.005219	KRT80	12	52168996
ENSG0000(	1546.003	1304.397	0.245579	6.17E-05	0.006115	CTGF	6	1.32E+08
ENSG0000(	929.5585	1132.398	-0.28455	6.73E-05	0.006597	INSIG2	2	1.18E+08
ENSG0000(	1931.946	2220.615	-0.20052	8.09E-05	0.007839	SFXN3	10	1.01E+08
ENSG0000(	410.6034	530.1137	-0.36833	9.64E-05	0.00919	RAB26	16	2140803
ENSG0000(	5157.475	5689.885	-0.14149	9.70E-05	0.00919	SERPINE2	2	2.24E+08
ENSG0000(	3019.581	3373.383	-0.15955	0.00011	0.010207	CRKL	22	20917426
ENSG0000(	4430.099	4009.88	0.143681	0.00011	0.010207	HEG1	3	1.25E+08
ENSG0000(	2137.743	2427.355	-0.18296	0.000112	0.010288	ERO1A	14	52639916
ENSG0000(	2348.484	2658.338	-0.17831	0.000119	0.010822	PPDPF	20	63520724
ENSG0000(	1155.878	1367.345	-0.24289	0.000128	0.011504	ANKZF1	2	2.19E+08
ENSG0000(	5956.881	5411.417	0.138752	0.00013	0.01151	MAT2A	2	85539165
ENSG0000(	1429.751	1665.595	-0.21969	0.000132	0.011568	RIOK3	18	23452823
ENSG0000(	2015.792	2285.465	-0.18132	0.00016	0.013866	WDR45B	17	82614562
ENSG0000(	770.9344	929.6955	-0.27038	0.00017	0.01466	PPP1R3B	8	9136255
ENSG0000(	4563.713	5067.246	-0.1507	0.0002	0.01699	RPL36	19	5674947
ENSG0000(	31459.8	34300.79	-0.12464	0.000209	0.017651	FTH1	11	61959718
ENSG0000(	9475.746	10541.93	-0.15408	0.000214	0.017866	LRP1	12	57128493
ENSG0000(	1470.966	1271.557	0.210311	0.000228	0.018791	1-Mar	1	2.21E+08
ENSG0000(	1685.541	1924.069	-0.19118	0.00023	0.018791	TBC1D8	2	1.01E+08
ENSG0000(	1082.279	904.3023	0.260027	0.000239	0.019383	GOLGA4	3	37243177

ENSG0000(	1185.2	1398.143	-0.23802	0.00025	0.020074	EEF2K	16	22206282
ENSG0000(	800.0553	658.0348	0.281148	0.000292	0.023049	DUSP2	2	96143166
ENSG0000(	376.4024	276.7031	0.445868	0.000293	0.023049	BRCA2	13	32315474
ENSG0000(	2789.703	3110.874	-0.15772	0.000311	0.024305	ELF3	1	2.02E+08
ENSG0000(	739.1451	603.394	0.292659	0.000333	0.025776	POP4	19	29604017
ENSG0000(	5379.041	5839.616	-0.11863	0.000358	0.027452	SCARB1	12	1.25E+08
ENSG0000(	65626.84	70539.36	-0.10414	0.000369	0.027988	GAPDH	12	6533927
ENSG0000(	5693.701	6176.215	-0.11715	0.000379	0.028498	PTP4A2	1	31906421
ENSG0000(	3582.808	3246.78	0.142303	0.000397	0.029646	ABCC2	10	99782640
ENSG0000(	1366.544	1571.89	-0.20181	0.000414	0.03064	METRN	16	715115
ENSG0000(	706.2823	865.4679	-0.29195	0.000449	0.032942	BTG1	12	92140278
ENSG0000(	621.2334	754.9522	-0.28065	0.000463	0.033638	FBXO42	1	16246839
ENSG0000(	860.3574	1029.699	-0.26004	0.000476	0.034194	KCTD11	17	7351889
ENSG0000(	22998.97	21465.29	0.09964	0.000484	0.034194	HSPA8	11	1.23E+08
ENSG0000(	1096.739	1276.846	-0.21982	0.000486	0.034194	PHF21A	11	45929323
ENSG0000(	939.458	772.5349	0.283078	0.000487	0.034194	SAMD9	7	93099513
ENSG0000(	825.7771	982.8435	-0.25179	0.000492	0.034288	IRF7	11	612553
ENSG0000(	1556.282	1769.8	-0.18528	0.000503	0.034782	PYGL	14	50857891
ENSG0000(	973.7518	1137.384	-0.22423	0.000548	0.037158	RAB40C	16	589357
ENSG0000(	1550.55	1759.802	-0.18234	0.000554	0.037158	TGFBI	5	1.36E+08
ENSG0000(	1066.387	1237.792	-0.21501	0.000561	0.037158	RRAGA	9	19049395
ENSG0000(	28170.38	30005.67	-0.0911	0.000562	0.037158	FOLR1	11	72189558
ENSG0000(	386.414	487.2799	-0.33445	0.000564	0.037158	PCDH1	5	1.42E+08
ENSG0000(	4799.897	5286.176	-0.13897	0.000564	0.037158	S100A10	1	1.52E+08
ENSG0000(	1935.652	2203.242	-0.18717	0.000584	0.038158	SLCO4A1	20	62642445
ENSG0000(	2410.677	2127.29	0.180015	0.000608	0.039401	GATA2	3	1.28E+08
ENSG0000(	3110.124	3450.622	-0.15022	0.000655	0.042141	SYDE1	19	15107403
ENSG0000(	1380.412	1200.25	0.201653	0.000662	0.042298	TIAL1	10	1.2E+08
ENSG0000(	293.2003	383.1142	-0.38705	0.000673	0.042631	ZNF395	8	28345585
ENSG0000(	495.0307	611.0474	-0.30267	0.000683	0.042993	CA5B	X	15688830
ENSG0000(	563.0635	686.3506	-0.28552	0.000724	0.045188	AKAP12	6	1.51E+08
ENSG0000(	720.3458	858.0878	-0.25208	0.00074	0.04589	YOD1	1	2.07E+08
ENSG0000(	1334.181	1156.868	0.205659	0.000767	0.047177	TPM1	15	63042632

gene_end	gene_stra	gene_leng	gene_bio	gene_desc	tf_family
45921874	-	4571	protein_c	insulin l	-
12917937	+	8440	protein_c	G protein	-
8374373	+	2482	protein_c	angiopoie	-
8026308	-	4186	protein_c	ERBB rece	-
54419460	-	7577	protein_c	integrin	-
1.01E+08	+	3190	protein_c	serpin fa	-
14489349	+	8254	protein_c	solute ca	-
74893359	+	6064	protein_c	hexokinas	-
1.54E+08	+	5498	protein_c	solute ca	-
1.96E+08	-	7887	protein_c	transferr	-
2.15E+08	-	17315	protein_c	fibronect	-
15200981	-	9394	protein_c	notch 3	-
10307397	+	2718	protein_c	adrenomed	-
28577264	-	2928	protein_c	aldolase,	-
78129296	+	5512	protein_c	phosphogl	-
1.22E+08	+	3793	protein_c	family wi	-
6923698	+	4281	protein_c	enolase 2	-
1.32E+08	-	5325	protein_c	BCL2 inte	-
1.1E+08	+	4607	protein_c	MAX inter	bHLH
26505636	+	4307	protein_c	BCL2 inte	-
73096974	-	3093	protein_c	prolyl 4-	-
2664802	+	6129	protein_c	tweety fa	-
1.73E+08	-	6128	protein_c	stannioca	-
48562015	-	6107	protein_c	6-phospho	-
19149290	-	5086	protein_c	perilipin	-
2841098	+	13968	protein_c	SH3 domai	-
53094779	+	7910	protein_c	integrin	-
42959176	-	4724	protein_c	solute ca	-
1.46E+08	-	7646	protein_c	procollag	-
90229679	+	7006	protein_c	semaphori	-
61714640	-	20047	protein_c	aspartate	-
34462774	-	7359	protein_c	egl-9 fam	-
1601	+	954	Mt_rRNA	mitochond	-
1.22E+08	-	5547	protein_c	lysyl oxi	-
1345099	-	6557	protein_c	CLPTM1 li	-
57226449	+	2150	protein_c	neurexoph	-
85583962	+	2455	protein_c	cysteine	-
89111398	-	13215	protein_c	family wi	-
44968071	-	1295	protein_c	complemen	-
568457	-	2321	lincRNA	MIR210 ho	-
36453791	+	6012	protein_c	anillin a	-
91314547	-	16538	antisense	lung canc	-
23559794	-	1797	protein_c	inhibitor	bHLH
5153595	+	14716	protein_c	lysine de	-
41012565	+	11517	protein_c	CTP synth	-
75757818	+	6761	protein_c	integrin	-
73780251	+	4910	protein_c	fucosyltr	-
45146422	-	6507	protein_c	CUB domai	-
23425328	-	5707	protein_c	lysyl oxi	-
1.49E+08	-	3668	protein_c	transmemb	-
1.73E+08	+	14883	protein_c	pyruvate	-

4985323	+	3837	protein_	basic he	bHLH
6870948	+	2700	protein_	trioseph	-
41353911	-	3238	protein_	transfor	-
1.2E+08	-	8727	protein_	follista	-
18408425	+	6210	protein_	lactate	-
66977004	+	1187	protein_	chromoso	-
1.28E+08	+	2070	protein_	hypoxia	-
41484673	-	1639	protein_	endothel	-
86952910	+	11725	protein_	serine p	-
1.69E+08	+	2962	protein_	ATPase N	-
27315926	+	8602	protein_	WD repea	-
45226278	-	1813	protein_	transmem	-
72276036	+	2058	protein_	DNA dama	-
91383359	-	4934	protein_	arrestin	-
86492716	+	13423	protein_	lysine d	-
65466720	+	1772	transcri	Finkel-B	-
10172138	-	3057	protein_	oxidized	-
14774897	-	7019	protein_	family w	-
57240715	-	2806	protein_	NDUFA4,	-
92929331	-	10150	protein_	early en	-
37807436	+	2344	protein_	H1 histo	-
1.8E+08	+	9311	protein_	quiescin	-
68291267	-	4061	protein_	solute c	-
7411486	-	9953	protein_	Scm like	-
2.03E+08	+	9707	protein_	PTPRF in	-
29630751	+	7717	protein_	BTB doma	TF_bZIP
2.01E+08	+	8085	protein_	basic le	-
1.55E+08	+	2539	protein_	ephrin A	-
39462300	-	7717	protein_	DAB2, cl	-
34402413	+	10680	protein_	glucose-	-
31817946	+	2483	protein_	heat sho	-
52192000	-	4350	protein_	keratin	-
1.32E+08	-	2339	protein_	connecti	-
1.18E+08	+	3950	protein_	insulin	-
1.01E+08	+	4631	protein_	siderofl	-
2154165	+	2488	protein_	RAB26, m	-
2.24E+08	-	8891	protein_	serpin f	-
20953749	+	5325	protein_	CRK like	-
1.25E+08	-	9925	protein_	heart de	-
52695900	-	7548	protein_	endoplas	-
63522206	+	1094	protein_	pancreat	-
2.19E+08	+	6119	protein_	ankyrin	-
85545280	+	4487	protein_	methioni	-
23486603	+	5320	protein_	RIO kina	-
82648553	-	3542	protein_	WD repea	-
9151574	-	5776	protein_	protein	-
5691876	+	2272	protein_	ribosoma	-
61967660	-	2030	protein_	ferritin	-
57213351	+	20839	protein_	LDL rece	-
2.21E+08	+	9258	protein_	mitochon	-
1.01E+08	-	6030	protein_	TBC1 dom	-
37366751	+	10737	protein_	golgin A	-

22288732	+	8020	protein_(	eukaryot:	-
96145440	-	1932	protein_(	dual spe(	-
32400266	+	12273	protein_(	BRCA2, DN	-
2.02E+08	+	7191	protein_(	E74 like	ETS
29617237	+	6574	protein_(	POP4 hom(	-
1.25E+08	-	7741	protein_(	scavenger	-
6538374	+	2981	protein_(	glyceral(	-
31944856	-	6719	protein_(	protein 1	-
99852594	+	7837	protein_(	ATP bind:	-
719655	+	3692	protein_(	meteorin,	-
92145897	-	4854	protein_(	BTG anti-	-
16352454	-	8984	protein_(	F-box pro	-
7354944	+	3056	protein_(	potassium	-
1.23E+08	-	4849	protein_(	heat sho(	-
46121178	-	11190	protein_(	PHD finge	-
93118023	-	6855	protein_(	sterile a	-
615999	-	2628	protein_(	interfer(	IRF
50944736	-	4798	protein_(	glycogen	-
629272	+	4180	protein_(	RAB40C, r	-
1.36E+08	+	7810	protein_(	transforr	-
19050983	+	1589	protein_(	Ras relat	-
72196323	+	1648	protein_(	folate re	-
1.42E+08	-	5903	protein_(	protocadh	-
1.52E+08	-	1543	protein_(	S100 cal(	-
62685785	+	4051	protein_(	solute ca	-
1.28E+08	-	4459	protein_(	GATA bin(	zf-GATA
15114988	+	3965	protein_(	synapse (	-
1.2E+08	-	7355	protein_(	TIA1 cyt(	-
28402701	-	5819	protein_(	zinc fing	-
15788409	+	8520	protein_(	carbonic	-
1.51E+08	+	8741	protein_(	A-kinase	-
2.07E+08	-	6521	protein_(	YOD1 deub	-
63071915	+	12876	protein_(	tropomyos	-

Accession	Gene Name
Q99613	EIF3C
Q8TAX9	GSDMB
P34931	HSPA1L
Q05193	DNM1
Q6UN15	FIP1L1
P30443	HLA-A
Q9BZQ8	FAM129A
Q05639	EEF1A2
Q86U42	PABPN1
Q10570	CPSF1
P32322	PYCR1
P40763	STAT3
P05166	PCCB
Q02750	MAP2K1
Q8WXI9	GATAD2B
Q15366	PCBP2
P26232	CTNNA2
Q14C86	GAPVD1
P42345	MTOR
O94925	GLS
Q96KG9	SCYL1
Q96A08	HIST1H2BA
Q14573	ITPR3
P04049	RAF1
P56377	AP1S2
Q10567	AP1B1
Q9BVG9	PTDSS2
Q13619	CUL4A
P10398	ARAF
Q8WTW3	COG1
Q12789	GTF3C1
Q9NWT6	HIF1AN
Q9UBM7	DHCR7
O14617	AP3D1
Q14192	FHL2
Q14669	TRIP12
P53814	SMTN
Q6SZW1	SARM1
P50148	GNAQ
O75439	PMPCB
Q9UPU5	USP24
O43159	RRP8
Q96S44	TP53RK
Q92888	ARHGEF1
O75477	ERLIN1
P23634	ATP2B4
Q9P0V3	SH3BP4
P62195	PSMC5
P62899	RPL31
O43150	ASAP2
Q5VYK3	ECPAS

P01584	IL1B
P22694	PRKACB
P60891	PRPS1
Q70UQ0	IKBIP
P86790	CCZ1B
Q9H3S7	PTPN23
P00387	CYB5R3
P23284	PPIB
Q99567	NUP88
O43823	AKAP8
Q9H6R4	NOL6
P82675	MRPS5
Q99575	POP1
O75396	SEC22B
Q08AF3	SLFN5
O60518	RANBP6
Q9Y241	HIGD1A
Q9Y508	RNF114
Q8IZ83	ALDH16A1
P51532	SMARCA4
Q3ZCQ8	TIMM50
Q01130	SRSF2
Q9NVH1	DNAJC11
O15160	POLR1C
Q9UBD5	ORC3
Q9Y2T2	AP3M1
Q03001	DST
Q14566	MCM6
Q9NPF4	OSGEP
Q13501	SQSTM1
O14979	HNRNPDL
O75691	UTP20
Q92597	NDRG1
O15479	MAGEB2
O75792	RNASEH2A
Q15427	SF3B4
P42226	STAT6
Q9HCN4	GPN1
Q96P47	AGAP3
Q13164	MAPK7
Q96JG6	VPS50
P40937	RFC5
Q06203	PPAT
Q5SY16	NOL9
O14929	HAT1
Q9HBH0	RHOF
Q13177	PAK2
P09001	MRPL3
Q8NF37	LPCAT1
P10620	MGST1
P78357	CNTNAP1
P82650	MRPS22

Q8IYB3	SRRM1
Q13838	DDX39B
P50750	CDK9
Q14653	IRF3
P42695	NCAPD3
P24390	KDELR1
Q5BJH7	YIF1B
P49753	ACOT2
Q6P4A7	SFXN4
Q9NQ39	RPS10P5
Q9Y5S1	TRPV2
P28838	LAP3
O60488	ACSL4
Q99959	PKP2
O00425	IGF2BP3
Q96RQ3	MCCC1
Q9BPW8	NIPSNAP1
Q96GQ5	C16orf58
Q9Y3D0	FAM96B
Q15043	SLC39A14
Q9UPT5	EXOC7
O14879	IFIT3
P52294	KPNA1
P27797	CALR
Q96HW7	INTS4
Q68EM7	ARHGAP17
Q6ZSR9	
Q9Y3M8	STARD13
O95340	PAPSS2
Q86YV9	HPS6
P30530	AXL
Q9UIJ7	AK3
P50851	LRBA
Q8TB37	NUBPL
Q68CQ7	GLT8D1
Q9H4A6	GOLPH3
O00400	SLC33A1
P00492	HPRT1
P51003	PAPOLA
P00750	PLAT
P61619	SEC61A1
O43252	PAPSS1
Q9Y394	DHRS7
O00468	AGRN
Q9BZH6	WDR11
O94822	LTN1
Q6NUM9	RETSAT
Q7L1Q6	BZW1
O60884	DNAJA2
Q92896	GLG1
Q15907	RAB11B
Q8NB90	SPATA5

Q9UDR5	AASS
P53007	SLC25A1
Q13308	PTK7
Q92529	SHC3
Q8IZL8	PELP1
Q9Y6E2	BZW2
P38606	ATP6V1A
P49790	NUP153
Q86VR2	RETREG3
P56182	RRP1
P07741	APRT
Q8TEM1	NUP210
O60244	MED14
Q6ZRV2	FAM83H
Q16537	PPP2R5E
Q9P0K7	RAI14
Q63ZY3	KANK2
Q8NI60	COQ8A
Q93034	CUL5
Q52LJ0	FAM98B
P62714	PPP2CB
P30044	PRDX5
P13498	CYBA
Q96KA5	CLPTM1L
Q9C0B5	ZDHHC5
Q4V328	GRIPAP1
P49321	NASP
Q9C0C9	UBE2O
P09496	CLTA
P40925	MDH1
O75955	FLOT1
O15260	SURF4
Q6ZVL6	KIAA1549L
Q567U6	CCDC93
Q96GK7	FAHD2A
Q02543	RPL18A
Q9C0E2	XPO4
Q04837	SSBP1
Q7Z4V5	HDGFL2
Q7Z2Z2	EFL1
Q6PI48	DARS2
Q8IYI6	EXOC8
Q32P28	P3H1
Q9Y2G5	POFUT2
Q27J81	INF2
Q9NZ01	TECR
Q96JH7	VCPIP1
Q15637	SF1
Q9NY33	DPP3
Q15042	RAB3GAP1
Q92804	TAF15
Q96JM3	CHAMP1

Q9UI12	ATP6V1H
P52907	CAPZA1
Q14195	DPYSL3
P22570	FDXR
Q5T9L3	WLS
P24941	CDK2
Q96ME1	FBXL18
Q9UNY4	TTF2
Q13315	ATM
P56385	ATP5ME
P62136	PPP1CA
Q9HD20	ATP13A1
Q9Y673	ALG5
Q13098	GPS1
Q9H8M5	CNNM2
Q5T1M5	FKBP15
Q5JSH3	WDR44
Q9NU22	MDN1
Q13601	KRR1
Q6XZF7	DNMBP
P62910	RPL32
Q99797	MIPEP
Q9BT22	ALG1
O75376	NCOR1
Q96TC7	RMDN3
Q9NUQ8	ABCF3
Q9H4A3	WNK1
Q9NUL3	STAU2
Q969E2	SCAMP4
P61964	WDR5
O00170	AIP
Q8IWV8	UBR2
P14923	JUP
P42685	FRK
Q00587	CDC42EP1
P49643	PRIM2
Q9P2K8	EIF2AK4
Q9NZJ4	SACS
Q7Z3C6	ATG9A
P28300	LOX
Q5T5P2	KIAA1217
O94804	STK10
Q12965	MYO1E
Q01968	OCRL
Q9NPD3	EXOSC4
Q8NBX0	SCCPDH
Q9BXI6	TBC1D10A
O15400	STX7
P00568	AK1
Q05086	UBE3A
Q8NBM4	UBAC2
Q9BZF1	OSBPL8

Q9Y5Y2	NUBP2
Q9HCS7	XAB2
P24666	ACP1
Q6ZSZ5	ARHGEF18
Q9Y3Z3	SAMHD1
Q8IZH2	XRN1
Q86TI2	DPP9
Q969N2	PIGT
O95865	DDAH2
Q7Z460	CLASP1
Q6IA86	ELP2
Q8WVB6	CHTF18
Q9UKG1	APPL1
P27144	AK4
P20337	RAB3B
A1L3X0	ELOVL7
Q9H9A5	CNOT10
Q9H6U8	ALG9
P61421	ATP6V0D1
Q8TAT6	NPLOC4
P63151	PPP2R2A
Q8NEM2	SHCBP1
P09012	SNRPA
P13473	LAMP2
Q8IUD2	ERC1
O43772	SLC25A20
Q13555	CAMK2G
O14545	TRAFD1
P23468	PTPRD
Q14738	PPP2R5D
Q96PU5	NEDD4L
P27361	MAPK3
P17252	PRKCA
O75843	AP1G2
P82930	MRPS34
Q14137	BOP1
P53350	PLK1
Q9Y2Z0	SUGT1
Q9NSE4	IARS2
Q86WR7	PROSER2
Q13418	ILK
Q9H267	VPS33B
O15357	INPPL1
Q15642	TRIP10
O75815	BCAR3
Q9H2V7	SPNS1
P84095	RHOG
Q9GZT9	EGLN1
Q66K74	MAP1S
O96000	NDUFB10
Q68DK2	ZFYVE26
Q9H1Y0	ATG5

Q9UJU6	DBNL
Q16851	UGP2
Q8TED1	GPX8
Q86T24	ZBTB33
O94915	FRYL
Q969Z0	TBRG4
P38935	IGHMBP2
P05106	ITGB3
P46379	BAG6
Q9BYN8	MRPS26
Q9Y240	CLEC11A
Q53GS9	USP39
O75822	EIF3J
Q96NW4	ANKRD27
Q969P0	IGSF8
P21912	SDHB
Q05048	CSTF1
Q9NZN3	EHD3
Q6IN85	PPP4R3A
Q8N6R0	METTL13
P36021	SLC16A2
Q5SRE5	NUP188
Q9Y6K5	OAS3
P01033	TIMP1
P52948	NUP98
Q9NX63	CHCHD3
Q86WJ1	CHD1L
P46734	MAP2K3
Q96EP0	RNF31
Q9Y399	MRPS2
Q5JTV8	TOR1AIP1
Q13136	PPFIA1
Q9Y2W6	TDRKH
Q96LT7	C9orf72
P78345	RPP38
Q9UI43	MRM2
Q9UQ90	SPG7
Q8TEQ6	GEMIN5
O43295	SRGAP3
Q2VPK5	CTU2
P49755	TMED10
Q9Y276	BCS1L
P54709	ATP1B3
Q7L7X3	TAOK1
Q9UNN5	FAF1
O95861	BPNT1
Q6NVY1	HIBCH
P60866	RPS20
P62854	RPS26
P10155	TROVE2
Q96D46	NMD3
Q9Y3U8	RPL36

O15511	ARPC5
P30566	ADSL
P62330	ARF6
P40616	ARL1
Q9Y4W6	AFG3L2
Q9Y2D5	AKAP2
C9JRZ8	AKR1B15
Q10469	MGAT2
Q16706	MAN2A1
Q9NP70	AMBN
Q5T2E6	ARMH3
P08243	ASNS
Q8NBU5	ATAD1
O75027	ABCB7
Q8WXE1	ATRIP
Q9NR09	BIRC6
Q9UBV7	B4GALT7
Q9UHR4	BAIAP2L1
Q6PJG6	BRAT1
Q9Y6D5	ARFGEF2
Q86YS7	C2CD5
O75844	ZMPSTE24
Q9HB71	CACYBP
Q8NCH0	CHST14
P50416	CPT1A
P48729	CSNK1A1
Q969G5	CAVIN3
Q96Q11	TRNT1
Q6YHK3	CD109
Q9H3Q1	CDC42EP4
Q13042	CDC16
Q9HD42	CHMP1A
Q8N684	CPSF7
P01024	C3
P29279	CTGF
Q9UP83	COG5
Q92478	CLEC2B
Q9Y6G9	DYNC1LI1
Q96KP4	CNDP2
Q86X76	NIT1
O94850	DDN
P25685	DNAJB1
O60762	DPM1
Q9UJW0	DCTN4
Q9UPN9	TRIM33
Q9HC35	EML4
Q7Z2K6	ERMP1
Q6P2E9	EDC4
Q9NTX5	ECHDC1
Q9UBQ5	EIF3K
P56537	EIF6
Q9NV70	EXOC1

Q9BXW9	FANCD2
Q9NVI1	FANCI
P51648	ALDH3A2
Q9UKT8	FBXW2
P23142	FBLN1
P51116	FXR2
P19447	ERCC3
P18074	ERCC2
Q6IA69	NADSYN1
P41250	GARS
P49841	GSK3B
P0CG08	GPR89B
Q8TBA6	GOLGA5
Q14393	GAS6
P09341	CXCL1
Q96HU8	DIRAS2
Q6AI08	HEATR6
Q9BYK8	HELZ2
Q9NR48	ASH1L
Q86TU7	SETD3
P42858	HTT
Q14520	HABP2
Q6PEW0	PRSS54
Q8IWB1	ITPRIP
Q9NX62	IMPAD1
Q9NQX7	ITM2C
Q9NVR2	INTS10
Q96CB8	INTS12
A0AVF1	TTC26
Q9UKU7	ACAD8
Q9BZD4	NUF2
Q8NBT2	SPC24
Q9UK59	DBR1
Q14766	LTBP1
Q6P1Q0	LETMD1
Q5T7N2	L1TD1
Q9H6V9	LDAH
Q6P1A2	LPCAT3
Q8NDA8	MROH1
P04732	MT1E
Q99707	MTR
Q02252	ALDH6A1
Q9NZJ7	MTCH1
O43615	TIMM44
Q15070	OXA1L
Q10713	PMPCA
O15264	MAPK13
Q13257	MAD2L1
Q13615	MTMR3
O95248	SBF1
Q86W25	NLRP13
Q6N069	NAA16

Q147X3	NAA30
P21359	NF1
O60934	NBN
P35658	NUP214
P51843	NR0B1
Q9BZE4	GTPBP4
Q9NTK5	OLA1
Q5JVF3	PCID2
P46019	PHKA2
Q53GA4	PHLDA2
P20742	PZP
Q5JRX3	PITRM1
O76071	CIAO1
P28370	SMARCA1
Q15031	LARS2
Q9HCU5	PREB
Q9H939	PSTPIP2
Q8IVL5	P3H2
Q15185	PTGES3
Q92530	PSMF1
P49720	PSMB3
Q96IU4	ABHD14B
Q13948	CUX1
Q9UGV2	NDRG3
Q8NHV4	NEDD1
Q96PU8	QKI
O15258	RER1
P06454	PTMA
Q6P996	PDXDC1
Q53H96	PYCR3
Q14699	RFTN1
Q86X10	RALGAPB
Q6R327	RICTOR
P20936	RASA1
Q15303	ERBB4
Q92900	UPF1
Q8WUF5	PPP1R13L
Q9NQC3	RTN4
Q8N1W1	ARHGEF28
Q14155	ARHGEF7
Q7LG56	RRM2B
A6NJZ7	RIMBP3C
Q9H0A0	NAT10
Q9BWH6	RPAP1
Q8WXA3	RUFY2
O43236	SEPT4
Q9UQ35	SRRM2
Q08170	SRSF4
Q16629	SRSF7
Q8N1F8	STK11IP
Q8IWU2	LMTK2
P51955	NEK2

Q8TDX7	NEK7
Q9UPN7	PPP6R1
Q96HS1	PGAM5
Q15165	PON2
Q8NEF9	SRFBP1
P45954	ACADSB
P52630	STAT2
Q96AG3	SLC25A46
Q13596	SNX1
Q9UNH6	SNX7
P28290	SSFA2
Q9NXE4	SMPD4
P26368	U2AF2
P31948	STIP1
Q9H4L7	SMARCAD1
Q66K14	TBC1D9B
Q9HBL0	TNS1
Q5VYS8	TUT7
Q96J01	THOC3
Q8IVF5	TIAM2
O14776	TCERG1
Q15631	TSN
Q99442	SEC62
P43307	SSR1
Q6ZXV5	TMTC3
Q7Z7H5	TMED4
Q9BVK6	TMED9
Q8NBN3	TMEM87A
P52333	JAK3
P54577	YARS
P54578	USP14
Q9UHP3	USP25
Q14139	UBE4A
P61086	UBE2K
P05161	ISG15
Q15386	UBE3C
Q3KQV9	UAP1L1
Q9HB07	C12orf10
Q9BZX2	UCK2
Q9UN37	VPS4A
P50552	VASP
Q8TDW0	LRRC8C
Q9Y3C0	WASHC3
Q2M389	WASHC4
Q9UNX4	WDR3
Q15061	WDR43
Q7Z739	YTHDF3
O75152	ZC3H11A
Q6NZY4	ZCCHC8
Q14202	ZMYM3
Q969S3	ZNF622
Q92785	DPF2

A7E2V4	ZSWIM8
Q8N1S5	SLC39A11

Description	Coverage
Eukaryotic translation initiation factor 3 subunit C OS=Homo sapiens OX=9606 GN=EIF3	9.200438
Gasdermin-B OS=Homo sapiens OX=9606 GN=GSDMB PE=1 SV=2	14.59854
Heat shock 70 kDa protein 1-like OS=Homo sapiens OX=9606 GN=HSPA1L PE=1 SV=2	12.4805
Dynamin-1 OS=Homo sapiens OX=9606 GN=DNM1 PE=1 SV=2	5.902778
Pre-mRNA 3'-end-processing factor FIP1 OS=Homo sapiens OX=9606 GN=FIP1L1 PE=1 SV	7.744108
HLA class I histocompatibility antigen, A-1 alpha chain OS=Homo sapiens OX=9606 GN=	12.05479
Protein Niban OS=Homo sapiens OX=9606 GN=FAM129A PE=1 SV=1	4.849138
Elongation factor 1-alpha 2 OS=Homo sapiens OX=9606 GN=EEF1A2 PE=1 SV=1	8.855292
Polyadenylate-binding protein 2 OS=Homo sapiens OX=9606 GN=PABPN1 PE=1 SV=3	5.228758
Cleavage and polyadenylation specificity factor subunit 1 OS=Homo sapiens OX=9606 G	1.178101
Pyrroline-5-carboxylate reductase 1, mitochondrial OS=Homo sapiens OX=9606 GN=PY	14.42006
Signal transducer and activator of transcription 3 OS=Homo sapiens OX=9606 GN=STAT	1.558442
Propionyl-CoA carboxylase beta chain, mitochondrial OS=Homo sapiens OX=9606 GN=I	2.96846
Dual specificity mitogen-activated protein kinase kinase 1 OS=Homo sapiens OX=9606 G	11.70483
Transcriptional repressor p66-beta OS=Homo sapiens OX=9606 GN=GATAD2B PE=1 SV=	7.419899
Poly(rC)-binding protein 2 OS=Homo sapiens OX=9606 GN=PCBP2 PE=1 SV=1	15.34247
Catenin alpha-2 OS=Homo sapiens OX=9606 GN=CTNNA2 PE=1 SV=5	1.783841
GTPase-activating protein and VPS9 domain-containing protein 1 OS=Homo sapiens OX	3.518268
Serine/threonine-protein kinase mTOR OS=Homo sapiens OX=9606 GN=MTOR PE=1 SV=	1.216163
Glutaminase kidney isoform, mitochondrial OS=Homo sapiens OX=9606 GN=GLS PE=1 S	3.587444
N-terminal kinase-like protein OS=Homo sapiens OX=9606 GN=SCYL1 PE=1 SV=1	6.435644
Histone H2B type 1-A OS=Homo sapiens OX=9606 GN=HIST1H2BA PE=1 SV=3	7.086614
Inositol 1,4,5-trisphosphate receptor type 3 OS=Homo sapiens OX=9606 GN=ITPR3 PE=	2.882815
RAF proto-oncogene serine/threonine-protein kinase OS=Homo sapiens OX=9606 GN=R	5.246914
AP-1 complex subunit sigma-2 OS=Homo sapiens OX=9606 GN=AP1S2 PE=1 SV=1	10.19108
AP-1 complex subunit beta-1 OS=Homo sapiens OX=9606 GN=AP1B1 PE=1 SV=2	6.954689
Phosphatidylserine synthase 2 OS=Homo sapiens OX=9606 GN=PTDSS2 PE=1 SV=1	4.517454
Cullin-4A OS=Homo sapiens OX=9606 GN=CUL4A PE=1 SV=3	2.635046
Serine/threonine-protein kinase A-Raf OS=Homo sapiens OX=9606 GN=ARAF PE=1 SV=2	3.465347
Conserved oligomeric Golgi complex subunit 1 OS=Homo sapiens OX=9606 GN=COG1 P	1.632653
General transcription factor 3C polypeptide 1 OS=Homo sapiens OX=9606 GN=GTF3C1	0.995733
Hypoxia-inducible factor 1-alpha inhibitor OS=Homo sapiens OX=9606 GN=HIF1AN PE=	4.584527
7-dehydrocholesterol reductase OS=Homo sapiens OX=9606 GN=DHCR7 PE=1 SV=1	6.315789
AP-3 complex subunit delta-1 OS=Homo sapiens OX=9606 GN=AP3D1 PE=1 SV=1	2.515178
Four and a half LIM domains protein 2 OS=Homo sapiens OX=9606 GN=FHL2 PE=1 SV=3	14.33692
E3 ubiquitin-protein ligase TRIP12 OS=Homo sapiens OX=9606 GN=TRIP12 PE=1 SV=1	0.853414
Smoothelin OS=Homo sapiens OX=9606 GN=SMTN PE=1 SV=7	3.380589
Sterile alpha and TIR motif-containing protein 1 OS=Homo sapiens OX=9606 GN=SARM1	2.209945
Guanine nucleotide-binding protein G(q) subunit alpha OS=Homo sapiens OX=9606 GN	9.192201
Mitochondrial-processing peptidase subunit beta OS=Homo sapiens OX=9606 GN=PMP	7.566462
Ubiquitin carboxyl-terminal hydrolase 24 OS=Homo sapiens OX=9606 GN=USP24 PE=1	1.335878
Ribosomal RNA-processing protein 8 OS=Homo sapiens OX=9606 GN=RRP8 PE=1 SV=2	3.508772
EKC/KEOPS complex subunit TP53RK OS=Homo sapiens OX=9606 GN=TP53RK PE=1 SV=	8.300395
Rho guanine nucleotide exchange factor 1 OS=Homo sapiens OX=9606 GN=ARHGEF1 PE	5.482456
Erlin-1 OS=Homo sapiens OX=9606 GN=ERLIN1 PE=1 SV=1	12.42775
Plasma membrane calcium-transporting ATPase 4 OS=Homo sapiens OX=9606 GN=ATP2	4.43191
SH3 domain-binding protein 4 OS=Homo sapiens OX=9606 GN=SH3BP4 PE=1 SV=1	1.142264
26S proteasome regulatory subunit 8 OS=Homo sapiens OX=9606 GN=PSMC5 PE=1 SV=	17.9803
60S ribosomal protein L31 OS=Homo sapiens OX=9606 GN=RPL31 PE=1 SV=1	8
Arf-GAP with SH3 domain, ANK repeat and PH domain-containing protein 2 OS=Homo s	2.485089
Proteasome adapter and scaffold protein ECM29 OS=Homo sapiens OX=9606 GN=ECPAS	1.95122

Interleukin-1 beta OS=Homo sapiens OX=9606 GN=IL1B PE=1 SV=2	8.178439
cAMP-dependent protein kinase catalytic subunit beta OS=Homo sapiens OX=9606 GN=	4.843305
Ribose-phosphate pyrophosphokinase 1 OS=Homo sapiens OX=9606 GN=PRPS1 PE=1 SV	11.94969
Inhibitor of nuclear factor kappa-B kinase-interacting protein OS=Homo sapiens OX=96(	6.571429
Vacuolar fusion protein CCZ1 homolog B OS=Homo sapiens OX=9606 GN=CCZ1B PE=1 S'	3.526971
Tyrosine-protein phosphatase non-receptor type 23 OS=Homo sapiens OX=9606 GN=PT	1.528117
NADH-cytochrome b5 reductase 3 OS=Homo sapiens OX=9606 GN=CYB5R3 PE=1 SV=3	7.973422
Peptidyl-prolyl cis-trans isomerase B OS=Homo sapiens OX=9606 GN=PPIB PE=1 SV=2	6.018519
Nuclear pore complex protein Nup88 OS=Homo sapiens OX=9606 GN=NUP88 PE=1 SV=:	2.42915
A-kinase anchor protein 8 OS=Homo sapiens OX=9606 GN=AKAP8 PE=1 SV=1	2.890173
Nucleolar protein 6 OS=Homo sapiens OX=9606 GN=NOL6 PE=1 SV=2	1.308901
28S ribosomal protein S5, mitochondrial OS=Homo sapiens OX=9606 GN=MRPS5 PE=1	5.116279
Ribonucleases P/MRP protein subunit POP1 OS=Homo sapiens OX=9606 GN=POP1 PE=1	1.171875
Vesicle-trafficking protein SEC22b OS=Homo sapiens OX=9606 GN=SEC22B PE=1 SV=4	8.837209
Schlafen family member 5 OS=Homo sapiens OX=9606 GN=SLFN5 PE=1 SV=1	3.030303
Ran-binding protein 6 OS=Homo sapiens OX=9606 GN=RANBP6 PE=1 SV=2	1.357466
HIG1 domain family member 1A, mitochondrial OS=Homo sapiens OX=9606 GN=HIGD1	19.35484
E3 ubiquitin-protein ligase RNF114 OS=Homo sapiens OX=9606 GN=RNF114 PE=1 SV=1	8.77193
Aldehyde dehydrogenase family 16 member A1 OS=Homo sapiens OX=9606 GN=ALDH1(	1.995012
Transcription activator BRG1 OS=Homo sapiens OX=9606 GN=SMARCA4 PE=1 SV=2	2.125076
Mitochondrial import inner membrane translocase subunit TIM50 OS=Homo sapiens O.	7.932011
Serine/arginine-rich splicing factor 2 OS=Homo sapiens OX=9606 GN=SRSF2 PE=1 SV=4	11.76471
DnaJ homolog subfamily C member 11 OS=Homo sapiens OX=9606 GN=DNAJC11 PE=1 S	5.545617
DNA-directed RNA polymerases I and III subunit RPAC1 OS=Homo sapiens OX=9606 GN=	4.624277
Origin recognition complex subunit 3 OS=Homo sapiens OX=9606 GN=ORC3 PE=1 SV=1	1.828411
AP-3 complex subunit mu-1 OS=Homo sapiens OX=9606 GN=AP3M1 PE=1 SV=1	8.133971
Dystonin OS=Homo sapiens OX=9606 GN=DST PE=1 SV=4	0.198151
DNA replication licensing factor MCM6 OS=Homo sapiens OX=9606 GN=MCM6 PE=1 SV:	1.339829
Probable tRNA N6-adenosine threonylcarbamoyltransferase OS=Homo sapiens OX=960(	5.074627
Sequestosome-1 OS=Homo sapiens OX=9606 GN=SQSTM1 PE=1 SV=1	5.909091
Heterogeneous nuclear ribonucleoprotein D-like OS=Homo sapiens OX=9606 GN=HNRN	6.190476
Small subunit processome component 20 homolog OS=Homo sapiens OX=9606 GN=UTI	1.292639
Protein NDRG1 OS=Homo sapiens OX=9606 GN=NDRG1 PE=1 SV=1	3.553299
Melanoma-associated antigen B2 OS=Homo sapiens OX=9606 GN=MAGEB2 PE=1 SV=3	7.210031
Ribonuclease H2 subunit A OS=Homo sapiens OX=9606 GN=RNASEH2A PE=1 SV=2	5.685619
Splicing factor 3B subunit 4 OS=Homo sapiens OX=9606 GN=SF3B4 PE=1 SV=1	3.301887
Signal transducer and activator of transcription 6 OS=Homo sapiens OX=9606 GN=STAT(	2.007084
GPN-loop GTPase 1 OS=Homo sapiens OX=9606 GN=GPN1 PE=1 SV=1	4.278075
Arf-GAP with GTPase, ANK repeat and PH domain-containing protein 3 OS=Homo sapien	1.828571
Mitogen-activated protein kinase 7 OS=Homo sapiens OX=9606 GN=MAPK7 PE=1 SV=2	2.573529
Syndetin OS=Homo sapiens OX=9606 GN=VPS50 PE=1 SV=3	1.556017
Replication factor C subunit 5 OS=Homo sapiens OX=9606 GN=RFC5 PE=1 SV=1	7.058824
Amidophosphoribosyltransferase OS=Homo sapiens OX=9606 GN=PPAT PE=1 SV=1	3.675048
Polynucleotide 5'-hydroxyl-kinase NOL9 OS=Homo sapiens OX=9606 GN=NOL9 PE=1 SV:	1.139601
Histone acetyltransferase type B catalytic subunit OS=Homo sapiens OX=9606 GN=HAT1	3.818616
Rho-related GTP-binding protein RhoF OS=Homo sapiens OX=9606 GN=RHOF PE=1 SV=1	8.056872
Serine/threonine-protein kinase PAK 2 OS=Homo sapiens OX=9606 GN=PAK2 PE=1 SV=3	3.053435
39S ribosomal protein L3, mitochondrial OS=Homo sapiens OX=9606 GN=MRPL3 PE=1	2.011494
Lysophosphatidylcholine acyltransferase 1 OS=Homo sapiens OX=9606 GN=LPCAT1 PE=	3.745318
Microsomal glutathione S-transferase 1 OS=Homo sapiens OX=9606 GN=MGST1 PE=1 SV	8.387097
Contactin-associated protein 1 OS=Homo sapiens OX=9606 GN=CNTNAP1 PE=1 SV=1	1.806358
28S ribosomal protein S22, mitochondrial OS=Homo sapiens OX=9606 GN=MRPS22 PE:	3.055556

Serine/arginine repetitive matrix protein 1 OS=Homo sapiens OX=9606 GN=SRRM1 PE=:	1.769912
Spliceosome RNA helicase DDX39B OS=Homo sapiens OX=9606 GN=DDX39B PE=1 SV=1	7.476636
Cyclin-dependent kinase 9 OS=Homo sapiens OX=9606 GN=CDK9 PE=1 SV=3	12.09677
Interferon regulatory factor 3 OS=Homo sapiens OX=9606 GN=IRF3 PE=1 SV=1	3.278689
Condensin-2 complex subunit D3 OS=Homo sapiens OX=9606 GN=NCAPD3 PE=1 SV=2	0.867824
ER lumen protein-retaining receptor 1 OS=Homo sapiens OX=9606 GN=KDELR1 PE=1 SV	3.773585
Protein YIF1B OS=Homo sapiens OX=9606 GN=YIF1B PE=1 SV=1	4.140127
Acyl-coenzyme A thioesterase 2, mitochondrial OS=Homo sapiens OX=9606 GN=ACOT2	2.277433
Sideroflexin-4 OS=Homo sapiens OX=9606 GN=SFXN4 PE=1 SV=1	5.341246
Putative 40S ribosomal protein S10-like OS=Homo sapiens OX=9606 GN=RPS10P5 PE=5	12.5
Transient receptor potential cation channel subfamily V member 2 OS=Homo sapiens O)	4.319372
Cytosol aminopeptidase OS=Homo sapiens OX=9606 GN=LAP3 PE=1 SV=3	5.780347
Long-chain-fatty-acid--CoA ligase 4 OS=Homo sapiens OX=9606 GN=ACSL4 PE=1 SV=2	2.109705
Plakophilin-2 OS=Homo sapiens OX=9606 GN=PKP2 PE=1 SV=2	1.475596
Insulin-like growth factor 2 mRNA-binding protein 3 OS=Homo sapiens OX=9606 GN=IG	2.763385
Methylcrotonoyl-CoA carboxylase subunit alpha, mitochondrial OS=Homo sapiens OX=	1.793103
Protein NipSnap homolog 1 OS=Homo sapiens OX=9606 GN=NIPSNAP1 PE=1 SV=1	3.169014
RUS1 family protein C16orf58 OS=Homo sapiens OX=9606 GN=C16orf58 PE=1 SV=2	4.487179
Mitotic spindle-associated MMXD complex subunit MIP18 OS=Homo sapiens OX=9606	13.49693
Zinc transporter ZIP14 OS=Homo sapiens OX=9606 GN=SLC39A14 PE=1 SV=3	2.642276
Exocyst complex component 7 OS=Homo sapiens OX=9606 GN=EXOC7 PE=1 SV=3	2.312925
Interferon-induced protein with tetratricopeptide repeats 3 OS=Homo sapiens OX=960(	2.653061
Importin subunit alpha-5 OS=Homo sapiens OX=9606 GN=KPNA1 PE=1 SV=3	3.345725
Calreticulin OS=Homo sapiens OX=9606 GN=CALR PE=1 SV=1	4.556355
Integrator complex subunit 4 OS=Homo sapiens OX=9606 GN=INTS4 PE=1 SV=2	1.765317
Rho GTPase-activating protein 17 OS=Homo sapiens OX=9606 GN=ARHGAP17 PE=1 SV=:	1.362089
Uncharacterized protein FLJ45252 OS=Homo sapiens OX=9606 PE=2 SV=2	5.070423
StAR-related lipid transfer protein 13 OS=Homo sapiens OX=9606 GN=STARD13 PE=1 SV	0.718778
Bifunctional 3'-phosphoadenosine 5'-phosphosulfate synthase 2 OS=Homo sapiens OX=!	8.957655
Hermansky-Pudlak syndrome 6 protein OS=Homo sapiens OX=9606 GN=HPS6 PE=1 SV=:	2.451613
Tyrosine-protein kinase receptor UFO OS=Homo sapiens OX=9606 GN=AXL PE=1 SV=4	4.474273
GTP:AMP phosphotransferase AK3, mitochondrial OS=Homo sapiens OX=9606 GN=AK3	6.60793
Lipopolysaccharide-responsive and beige-like anchor protein OS=Homo sapiens OX=96(	1.327279
Iron-sulfur protein NUBPL OS=Homo sapiens OX=9606 GN=NUBPL PE=1 SV=3	6.269592
Glycosyltransferase 8 domain-containing protein 1 OS=Homo sapiens OX=9606 GN=GLT	3.773585
Golgi phosphoprotein 3 OS=Homo sapiens OX=9606 GN=GOLPH3 PE=1 SV=1	5.033557
Acetyl-coenzyme A transporter 1 OS=Homo sapiens OX=9606 GN=SLC33A1 PE=1 SV=1	4.918033
Hypoxanthine-guanine phosphoribosyltransferase OS=Homo sapiens OX=9606 GN=HPR	9.174312
Poly(A) polymerase alpha OS=Homo sapiens OX=9606 GN=PAPOLA PE=1 SV=4	1.47651
Tissue-type plasminogen activator OS=Homo sapiens OX=9606 GN=PLAT PE=1 SV=1	2.846975
Protein transport protein Sec61 subunit alpha isoform 1 OS=Homo sapiens OX=9606 GI	6.512605
Bifunctional 3'-phosphoadenosine 5'-phosphosulfate synthase 1 OS=Homo sapiens OX=!	5.929487
Dehydrogenase/reductase SDR family member 7 OS=Homo sapiens OX=9606 GN=DHRS7	3.244838
Agrin OS=Homo sapiens OX=9606 GN=AGRN PE=1 SV=5	1.257862
WD repeat-containing protein 11 OS=Homo sapiens OX=9606 GN=WDR11 PE=1 SV=1	0.980392
E3 ubiquitin-protein ligase listerin OS=Homo sapiens OX=9606 GN=LTN1 PE=1 SV=6	0.962627
All-trans-retinol 13,14-reductase OS=Homo sapiens OX=9606 GN=RETSAT PE=1 SV=2	2.131148
Basic leucine zipper and W2 domain-containing protein 1 OS=Homo sapiens OX=9606 (	5.966587
DnaJ homolog subfamily A member 2 OS=Homo sapiens OX=9606 GN=DNAJA2 PE=1 SV=	5.582524
Golgi apparatus protein 1 OS=Homo sapiens OX=9606 GN=GLG1 PE=1 SV=2	1.272265
Ras-related protein Rab-11B OS=Homo sapiens OX=9606 GN=RAB11B PE=1 SV=4	5.504587
Spermatogenesis-associated protein 5 OS=Homo sapiens OX=9606 GN=SPATA5 PE=1 SV=	2.68757

Alpha-aminoadipic semialdehyde synthase, mitochondrial OS=Homo sapiens OX=9606	0.971922
Tricarboxylate transport protein, mitochondrial OS=Homo sapiens OX=9606 GN=SLC25	6.109325
Inactive tyrosine-protein kinase 7 OS=Homo sapiens OX=9606 GN=PTK7 PE=1 SV=2	3.084112
SHC-transforming protein 3 OS=Homo sapiens OX=9606 GN=SHC3 PE=1 SV=1	2.525253
Proline-, glutamic acid- and leucine-rich protein 1 OS=Homo sapiens OX=9606 GN=PELF	1.858407
Basic leucine zipper and W2 domain-containing protein 2 OS=Homo sapiens OX=9606 G	5.48926
V-type proton ATPase catalytic subunit A OS=Homo sapiens OX=9606 GN=ATP6V1A PE=1	9.400324
Nuclear pore complex protein Nup153 OS=Homo sapiens OX=9606 GN=NUP153 PE=1 S	0.610169
Reticulophagy regulator 3 OS=Homo sapiens OX=9606 GN=RETREG3 PE=1 SV=1	4.077253
Ribosomal RNA processing protein 1 homolog A OS=Homo sapiens OX=9606 GN=RRP1 F	2.819957
Adenine phosphoribosyltransferase OS=Homo sapiens OX=9606 GN=APRT PE=1 SV=2	7.222222
Nuclear pore membrane glycoprotein 210 OS=Homo sapiens OX=9606 GN=NUP210 PE=	0.741918
Mediator of RNA polymerase II transcription subunit 14 OS=Homo sapiens OX=9606 GN	1.994498
Protein FAM83H OS=Homo sapiens OX=9606 GN=FAM83H PE=1 SV=3	1.357082
Serine/threonine-protein phosphatase 2A 56 kDa regulatory subunit epsilon isoform OS	2.783726
Ankycorbin OS=Homo sapiens OX=9606 GN=RAI14 PE=1 SV=2	1.22449
KN motif and ankyrin repeat domain-containing protein 2 OS=Homo sapiens OX=9606 G	4.700353
Atypical kinase COQ8A, mitochondrial OS=Homo sapiens OX=9606 GN=COQ8A PE=1 SV=	1.545595
Cullin-5 OS=Homo sapiens OX=9606 GN=CUL5 PE=1 SV=4	1.666667
Protein FAM98B OS=Homo sapiens OX=9606 GN=FAM98B PE=1 SV=1	3.636364
Serine/threonine-protein phosphatase 2A catalytic subunit beta isoform OS=Homo sapi	4.530744
Peroxiredoxin-5, mitochondrial OS=Homo sapiens OX=9606 GN=PRDX5 PE=1 SV=4	5.140187
Cytochrome b-245 light chain OS=Homo sapiens OX=9606 GN=CYBA PE=1 SV=3	5.128205
Cleft lip and palate transmembrane protein 1-like protein OS=Homo sapiens OX=9606 G	2.416357
Palmitoyltransferase ZDHHC5 OS=Homo sapiens OX=9606 GN=ZDHHC5 PE=1 SV=2	2.237762
GRIP1-associated protein 1 OS=Homo sapiens OX=9606 GN=GRIPAP1 PE=1 SV=1	1.307967
Nuclear autoantigenic sperm protein OS=Homo sapiens OX=9606 GN=NASP PE=1 SV=2	1.395939
(E3-independent) E2 ubiquitin-conjugating enzyme OS=Homo sapiens OX=9606 GN=UBI	1.083591
Clathrin light chain A OS=Homo sapiens OX=9606 GN=CLTA PE=1 SV=1	3.629032
Malate dehydrogenase, cytoplasmic OS=Homo sapiens OX=9606 GN=MDH1 PE=1 SV=4	5.08982
Flotillin-1 OS=Homo sapiens OX=9606 GN=FLOT1 PE=1 SV=3	2.107728
Surfeit locus protein 4 OS=Homo sapiens OX=9606 GN=SURF4 PE=1 SV=3	3.717472
UPF0606 protein KIAA1549L OS=Homo sapiens OX=9606 GN=KIAA1549L PE=2 SV=2	0.378583
Coiled-coil domain-containing protein 93 OS=Homo sapiens OX=9606 GN=CCDC93 PE=	1.584786
Fumarylacetoacetate hydrolase domain-containing protein 2A OS=Homo sapiens OX=96	3.503185
60S ribosomal protein L18a OS=Homo sapiens OX=9606 GN=RPL18A PE=1 SV=2	7.386364
Exportin-4 OS=Homo sapiens OX=9606 GN=XPO4 PE=1 SV=2	1.129453
Single-stranded DNA-binding protein, mitochondrial OS=Homo sapiens OX=9606 GN=S	10.13514
Hepatoma-derived growth factor-related protein 2 OS=Homo sapiens OX=9606 GN=HD	2.235469
Elongation factor-like GTPase 1 OS=Homo sapiens OX=9606 GN=EFL1 PE=1 SV=2	0.625
Aspartate--tRNA ligase, mitochondrial OS=Homo sapiens OX=9606 GN=DARS2 PE=1 SV=	3.100775
Exocyst complex component 8 OS=Homo sapiens OX=9606 GN=EXOC8 PE=1 SV=2	2.896552
Prolyl 3-hydroxylase 1 OS=Homo sapiens OX=9606 GN=P3H1 PE=1 SV=2	3.125
GDP-fucose protein O-fucosyltransferase 2 OS=Homo sapiens OX=9606 GN=POFUT2 PE=	2.564103
Inverted formin-2 OS=Homo sapiens OX=9606 GN=INF2 PE=1 SV=2	0.640512
Very-long-chain enoyl-CoA reductase OS=Homo sapiens OX=9606 GN=TECR PE=1 SV=1	5.519481
Deubiquitinating protein VCIP135 OS=Homo sapiens OX=9606 GN=VCPIP1 PE=1 SV=2	2.291326
Splicing factor 1 OS=Homo sapiens OX=9606 GN=SF1 PE=1 SV=4	2.190923
Dipeptidyl peptidase 3 OS=Homo sapiens OX=9606 GN=DPP3 PE=1 SV=2	6.377205
Rab3 GTPase-activating protein catalytic subunit OS=Homo sapiens OX=9606 GN=RAB3	1.325178
TATA-binding protein-associated factor 2N OS=Homo sapiens OX=9606 GN=TAF15 PE=1	1.689189
Chromosome alignment-maintaining phosphoprotein 1 OS=Homo sapiens OX=9606 GN	0.985222

V-type proton ATPase subunit H OS=Homo sapiens OX=9606 GN=ATP6V1H PE=1 SV=1	1.449275
F-actin-capping protein subunit alpha-1 OS=Homo sapiens OX=9606 GN=CAPZA1 PE=1 S	9.090909
Dihydropyrimidinase-related protein 3 OS=Homo sapiens OX=9606 GN=DPYSL3 PE=1 SV	4.035088
NADPH:adrenodoxin oxidoreductase, mitochondrial OS=Homo sapiens OX=9606 GN=FI	4.684318
Protein wntless homolog OS=Homo sapiens OX=9606 GN=WLS PE=1 SV=2	1.663586
Cyclin-dependent kinase 2 OS=Homo sapiens OX=9606 GN=CDK2 PE=1 SV=2	11.74497
F-box/LRR-repeat protein 18 OS=Homo sapiens OX=9606 GN=FBXL18 PE=1 SV=2	1.490683
Transcription termination factor 2 OS=Homo sapiens OX=9606 GN=TTF2 PE=1 SV=2	0.60241
Serine-protein kinase ATM OS=Homo sapiens OX=9606 GN=ATM PE=1 SV=4	0.78534
ATP synthase subunit e, mitochondrial OS=Homo sapiens OX=9606 GN=ATP5ME PE=1 SV	14.49275
Serine/threonine-protein phosphatase PP1-alpha catalytic subunit OS=Homo sapiens O	8.484848
Manganese-transporting ATPase 13A1 OS=Homo sapiens OX=9606 GN=ATP13A1 PE=1 SV	1.910299
Dolichyl-phosphate beta-glucosyltransferase OS=Homo sapiens OX=9606 GN=ALG5 PE=	3.703704
COP9 signalosome complex subunit 1 OS=Homo sapiens OX=9606 GN=GPS1 PE=1 SV=4	2.240326
Metal transporter CNNM2 OS=Homo sapiens OX=9606 GN=CNNM2 PE=1 SV=2	1.6
FK506-binding protein 15 OS=Homo sapiens OX=9606 GN=FKBP15 PE=1 SV=2	0.984413
WD repeat-containing protein 44 OS=Homo sapiens OX=9606 GN=WDR44 PE=1 SV=1	4.381161
Midasin OS=Homo sapiens OX=9606 GN=MDN1 PE=1 SV=2	0.125089
KRR1 small subunit processome component homolog OS=Homo sapiens OX=9606 GN=I	3.149606
Dynamin-binding protein OS=Homo sapiens OX=9606 GN=DNMBP PE=1 SV=1	0.887762
60S ribosomal protein L32 OS=Homo sapiens OX=9606 GN=RPL32 PE=1 SV=2	9.62963
Mitochondrial intermediate peptidase OS=Homo sapiens OX=9606 GN=MIPEP PE=1 SV=	2.945302
Chitobiosyldiphosphodolichol beta-mannosyltransferase OS=Homo sapiens OX=9606 G	4.094828
Nuclear receptor corepressor 1 OS=Homo sapiens OX=9606 GN=NCOR1 PE=1 SV=2	1.434426
Regulator of microtubule dynamics protein 3 OS=Homo sapiens OX=9606 GN=RMDN3 F	3.829787
ATP-binding cassette sub-family F member 3 OS=Homo sapiens OX=9606 GN=ABCF3 PE=	1.410437
Serine/threonine-protein kinase WNK1 OS=Homo sapiens OX=9606 GN=WNK1 PE=1 SV=	0.503778
Double-stranded RNA-binding protein Staufen homolog 2 OS=Homo sapiens OX=9606 G	2.280702
Secretory carrier-associated membrane protein 4 OS=Homo sapiens OX=9606 GN=SCAM	5.240175
WD repeat-containing protein 5 OS=Homo sapiens OX=9606 GN=WDR5 PE=1 SV=1	3.293413
AH receptor-interacting protein OS=Homo sapiens OX=9606 GN=AIP PE=1 SV=2	4.242424
E3 ubiquitin-protein ligase UBR2 OS=Homo sapiens OX=9606 GN=UBR2 PE=1 SV=1	0.740741
Junction plakoglobin OS=Homo sapiens OX=9606 GN=JUP PE=1 SV=3	1.610738
Tyrosine-protein kinase FRK OS=Homo sapiens OX=9606 GN=FRK PE=1 SV=1	1.584158
Cdc42 effector protein 1 OS=Homo sapiens OX=9606 GN=CDC42EP1 PE=1 SV=1	4.859335
DNA primase large subunit OS=Homo sapiens OX=9606 GN=PRIM2 PE=1 SV=2	2.946955
eIF-2-alpha kinase GCN2 OS=Homo sapiens OX=9606 GN=EIF2AK4 PE=1 SV=3	1.212856
Sacsin OS=Homo sapiens OX=9606 GN=SACS PE=1 SV=2	0.589648
Autophagy-related protein 9A OS=Homo sapiens OX=9606 GN=ATG9A PE=1 SV=3	1.668653
Protein-lysine 6-oxidase OS=Homo sapiens OX=9606 GN=LOX PE=1 SV=2	1.678657
Sickle tail protein homolog OS=Homo sapiens OX=9606 GN=KIAA1217 PE=1 SV=2	0.514668
Serine/threonine-protein kinase 10 OS=Homo sapiens OX=9606 GN=STK10 PE=1 SV=1	2.169421
Unconventional myosin-Ie OS=Homo sapiens OX=9606 GN=MYO1E PE=1 SV=2	1.083032
Inositol polyphosphate 5-phosphatase OCRL-1 OS=Homo sapiens OX=9606 GN=OCRL PE	1.220866
Exosome complex component RRP41 OS=Homo sapiens OX=9606 GN=EXOSC4 PE=1 SV=	4.897959
Saccharopine dehydrogenase-like oxidoreductase OS=Homo sapiens OX=9606 GN=SCCF	8.158508
TBC1 domain family member 10A OS=Homo sapiens OX=9606 GN=TBC1D10A PE=1 SV=1	1.377953
Syntaxin-7 OS=Homo sapiens OX=9606 GN=STX7 PE=1 SV=4	5.747126
Adenylate kinase isoenzyme 1 OS=Homo sapiens OX=9606 GN=AK1 PE=1 SV=3	5.670103
Ubiquitin-protein ligase E3A OS=Homo sapiens OX=9606 GN=UBE3A PE=1 SV=4	1.142857
Ubiquitin-associated domain-containing protein 2 OS=Homo sapiens OX=9606 GN=UB/	5.232558
Oxysterol-binding protein-related protein 8 OS=Homo sapiens OX=9606 GN=OSBPL8 PE	2.699663

Cytosolic Fe-S cluster assembly factor NUBP2 OS=Homo sapiens OX=9606 GN=NUBP2 PE	5.166052
Pre-mRNA-splicing factor SYF1 OS=Homo sapiens OX=9606 GN=XAB2 PE=1 SV=2	1.520468
Low molecular weight phosphotyrosine protein phosphatase OS=Homo sapiens OX=96(	11.39241
Rho guanine nucleotide exchange factor 18 OS=Homo sapiens OX=9606 GN=ARHGEF18	1.534527
Deoxynucleoside triphosphate triphosphohydrolase SAMHD1 OS=Homo sapiens OX=96	4.632588
5'-3' exoribonuclease 1 OS=Homo sapiens OX=9606 GN=XRN1 PE=1 SV=1	0.586166
Dipeptidyl peptidase 9 OS=Homo sapiens OX=9606 GN=DPP9 PE=1 SV=3	2.549247
GPI transamidase component PIG-T OS=Homo sapiens OX=9606 GN=PIGT PE=1 SV=1	4.32526
N(G),N(G)-dimethylarginine dimethylaminohydrolase 2 OS=Homo sapiens OX=9606 GN	11.22807
CLIP-associating protein 1 OS=Homo sapiens OX=9606 GN=CLASP1 PE=1 SV=1	0.845254
Elongator complex protein 2 OS=Homo sapiens OX=9606 GN=ELP2 PE=1 SV=2	1.089588
Chromosome transmission fidelity protein 18 homolog OS=Homo sapiens OX=9606 GN	2.461538
DCC-interacting protein 13-alpha OS=Homo sapiens OX=9606 GN=APPL1 PE=1 SV=1	1.974612
Adenylate kinase 4, mitochondrial OS=Homo sapiens OX=9606 GN=AK4 PE=1 SV=1	10.76233
Ras-related protein Rab-3B OS=Homo sapiens OX=9606 GN=RAB3B PE=1 SV=2	5.479452
Elongation of very long chain fatty acids protein 7 OS=Homo sapiens OX=9606 GN=ELOV	2.846975
CCR4-NOT transcription complex subunit 10 OS=Homo sapiens OX=9606 GN=CNOT10 F	1.88172
Alpha-1,2-mannosyltransferase ALG9 OS=Homo sapiens OX=9606 GN=ALG9 PE=1 SV=2	2.291326
V-type proton ATPase subunit d 1 OS=Homo sapiens OX=9606 GN=ATP6V0D1 PE=1 SV=1	5.128205
Nuclear protein localization protein 4 homolog OS=Homo sapiens OX=9606 GN=NPLOC	2.302632
Serine/threonine-protein phosphatase 2A 55 kDa regulatory subunit B alpha isoform OS	2.46085
SHC SH2 domain-binding protein 1 OS=Homo sapiens OX=9606 GN=SHCBP1 PE=1 SV=3	1.190476
U1 small nuclear ribonucleoprotein A OS=Homo sapiens OX=9606 GN=SNRPA PE=1 SV=:	2.48227
Lysosome-associated membrane glycoprotein 2 OS=Homo sapiens OX=9606 GN=LAMP2	1.95122
ELKS/Rab6-interacting/CAST family member 1 OS=Homo sapiens OX=9606 GN=ERC1 PE=	1.164875
Mitochondrial carnitine/acylcarnitine carrier protein OS=Homo sapiens OX=9606 GN=S	2.657807
Calcium/calmodulin-dependent protein kinase type II subunit gamma OS=Homo sapien	3.584229
TRAF-type zinc finger domain-containing protein 1 OS=Homo sapiens OX=9606 GN=TRA	1.718213
Receptor-type tyrosine-protein phosphatase delta OS=Homo sapiens OX=9606 GN=PTPI	0.523013
Serine/threonine-protein phosphatase 2A 56 kDa regulatory subunit delta isoform OS=H	1.993355
E3 ubiquitin-protein ligase NEDD4-like OS=Homo sapiens OX=9606 GN=NEDD4L PE=1 S\	2.153846
Mitogen-activated protein kinase 3 OS=Homo sapiens OX=9606 GN=MAPK3 PE=1 SV=4	8.17942
Protein kinase C alpha type OS=Homo sapiens OX=9606 GN=PRKCA PE=1 SV=4	2.678571
AP-1 complex subunit gamma-like 2 OS=Homo sapiens OX=9606 GN=AP1G2 PE=1 SV=1	2.292994
28S ribosomal protein S34, mitochondrial OS=Homo sapiens OX=9606 GN=MRPS34 PE=	3.669725
Ribosome biogenesis protein BOP1 OS=Homo sapiens OX=9606 GN=BOP1 PE=1 SV=2	1.340483
Serine/threonine-protein kinase PLK1 OS=Homo sapiens OX=9606 GN=PLK1 PE=1 SV=1	5.306799
Protein SGT1 homolog OS=Homo sapiens OX=9606 GN=SUGT1 PE=1 SV=3	3.287671
Isoleucine--tRNA ligase, mitochondrial OS=Homo sapiens OX=9606 GN=IARS2 PE=1 SV=:	4.051383
Proline and serine-rich protein 2 OS=Homo sapiens OX=9606 GN=PROSER2 PE=1 SV=2	2.298851
Integrin-linked protein kinase OS=Homo sapiens OX=9606 GN=ILK PE=1 SV=2	1.769912
Vacuolar protein sorting-associated protein 33B OS=Homo sapiens OX=9606 GN=VPS33	4.538088
Phosphatidylinositol 3,4,5-trisphosphate 5-phosphatase 2 OS=Homo sapiens OX=9606	1.112878
Cdc42-interacting protein 4 OS=Homo sapiens OX=9606 GN=TRIP10 PE=1 SV=3	5.15807
Breast cancer anti-estrogen resistance protein 3 OS=Homo sapiens OX=9606 GN=BCAR3	2.060606
Protein spinster homolog 1 OS=Homo sapiens OX=9606 GN=SPNS1 PE=1 SV=1	2.651515
Rho-related GTP-binding protein RhoG OS=Homo sapiens OX=9606 GN=RHOG PE=1 SV=	9.947644
Egl nine homolog 1 OS=Homo sapiens OX=9606 GN=EGLN1 PE=1 SV=1	2.58216
Microtubule-associated protein 1S OS=Homo sapiens OX=9606 GN=MAP1S PE=1 SV=2	1.605288
NADH dehydrogenase [ubiquinone] 1 beta subcomplex subunit 10 OS=Homo sapiens O)	6.395349
Zinc finger FYVE domain-containing protein 26 OS=Homo sapiens OX=9606 GN=ZFYVE2(	0.393856
Autophagy protein 5 OS=Homo sapiens OX=9606 GN=ATG5 PE=1 SV=2	3.636364

Drebrin-like protein OS=Homo sapiens OX=9606 GN=DBNL PE=1 SV=1	3.72093
UTP--glucose-1-phosphate uridylyltransferase OS=Homo sapiens OX=9606 GN=UGP2 PE	1.771654
Probable glutathione peroxidase 8 OS=Homo sapiens OX=9606 GN=GPX8 PE=1 SV=2	5.263158
Transcriptional regulator Kaiso OS=Homo sapiens OX=9606 GN=ZBTB33 PE=1 SV=2	1.190476
Protein furry homolog-like OS=Homo sapiens OX=9606 GN=FRYL PE=1 SV=2	0.298706
FAST kinase domain-containing protein 4 OS=Homo sapiens OX=9606 GN=TBRG4 PE=1 S	3.328051
DNA-binding protein SMUBP-2 OS=Homo sapiens OX=9606 GN=IGHMBP2 PE=1 SV=3	1.107754
Integrin beta-3 OS=Homo sapiens OX=9606 GN=ITGB3 PE=1 SV=2	2.030457
Large proline-rich protein BAG6 OS=Homo sapiens OX=9606 GN=BAG6 PE=1 SV=2	0.883392
28S ribosomal protein S26, mitochondrial OS=Homo sapiens OX=9606 GN=MRPS26 PE=	5.365854
C-type lectin domain family 11 member A OS=Homo sapiens OX=9606 GN=CLEC11A PE=	3.71517
U4/U6.U5 tri-snRNP-associated protein 2 OS=Homo sapiens OX=9606 GN=USP39 PE=1 S	1.946903
Eukaryotic translation initiation factor 3 subunit J OS=Homo sapiens OX=9606 GN=EIF3	3.100775
Ankyrin repeat domain-containing protein 27 OS=Homo sapiens OX=9606 GN=ANKRD2	1.52381
Immunoglobulin superfamily member 8 OS=Homo sapiens OX=9606 GN=IGSF8 PE=1 SV	2.28385
Succinate dehydrogenase [ubiquinone] iron-sulfur subunit, mitochondrial OS=Homo sa	6.071429
Cleavage stimulation factor subunit 1 OS=Homo sapiens OX=9606 GN=CSTF1 PE=1 SV=1	4.176334
EH domain-containing protein 3 OS=Homo sapiens OX=9606 GN=EHD3 PE=1 SV=2	3.925234
Serine/threonine-protein phosphatase 4 regulatory subunit 3A OS=Homo sapiens OX=96	1.080432
Methyltransferase-like protein 13 OS=Homo sapiens OX=9606 GN=METTL13 PE=1 SV=1	1.430615
Monocarboxylate transporter 8 OS=Homo sapiens OX=9606 GN=SLC16A2 PE=1 SV=2	2.040816
Nucleoporin NUP188 homolog OS=Homo sapiens OX=9606 GN=NUP188 PE=1 SV=1	0.400229
2'-5'-oligoadenylate synthase 3 OS=Homo sapiens OX=9606 GN=OAS3 PE=1 SV=3	2.115915
Metalloproteinase inhibitor 1 OS=Homo sapiens OX=9606 GN=TIMP1 PE=1 SV=1	5.797101
Nuclear pore complex protein Nup98-Nup96 OS=Homo sapiens OX=9606 GN=NUP98 PI	0.935608
MICOS complex subunit MIC19 OS=Homo sapiens OX=9606 GN=CHCHD3 PE=1 SV=1	5.286344
Chromodomain-helicase-DNA-binding protein 1-like OS=Homo sapiens OX=9606 GN=CH	3.010033
Dual specificity mitogen-activated protein kinase kinase 3 OS=Homo sapiens OX=9606 G	7.204611
E3 ubiquitin-protein ligase RNF31 OS=Homo sapiens OX=9606 GN=RNF31 PE=1 SV=1	1.212687
28S ribosomal protein S2, mitochondrial OS=Homo sapiens OX=9606 GN=MRPS2 PE=1	5.405405
Torsin-1A-interacting protein 1 OS=Homo sapiens OX=9606 GN=TOR1AIP1 PE=1 SV=2	2.058319
Liprin-alpha-1 OS=Homo sapiens OX=9606 GN=PPFIA1 PE=1 SV=1	0.915141
Tudor and KH domain-containing protein OS=Homo sapiens OX=9606 GN=TDRKH PE=1	2.139037
Guanine nucleotide exchange C9orf72 OS=Homo sapiens OX=9606 GN=C9orf72 PE=1 SV	2.286902
Ribonuclease P protein subunit p38 OS=Homo sapiens OX=9606 GN=RPP38 PE=1 SV=2	2.826855
rRNA methyltransferase 2, mitochondrial OS=Homo sapiens OX=9606 GN=MRM2 PE=1 S	3.252033
Paraplegin OS=Homo sapiens OX=9606 GN=SPG7 PE=1 SV=2	1.383648
Gem-associated protein 5 OS=Homo sapiens OX=9606 GN=GEMIN5 PE=1 SV=3	1.923077
SLIT-ROBO Rho GTPase-activating protein 3 OS=Homo sapiens OX=9606 GN=SRGAP3 PE=	0.818926
Cytoplasmic tRNA 2-thiolation protein 2 OS=Homo sapiens OX=9606 GN=CTU2 PE=1 SV	2.718447
Transmembrane emp24 domain-containing protein 10 OS=Homo sapiens OX=9606 GN=	9.13242
Mitochondrial chaperone BCS1 OS=Homo sapiens OX=9606 GN=BCS1L PE=1 SV=1	2.386635
Sodium/potassium-transporting ATPase subunit beta-3 OS=Homo sapiens OX=9606 GN=	3.942652
Serine/threonine-protein kinase TAO1 OS=Homo sapiens OX=9606 GN=TAOK1 PE=1 SV=1	1.298701
FAS-associated factor 1 OS=Homo sapiens OX=9606 GN=FAF1 PE=1 SV=2	1.692308
3'(2'),5'-bisphosphate nucleotidase 1 OS=Homo sapiens OX=9606 GN=BPNT1 PE=1 SV=1	2.597403
3-hydroxyisobutyryl-CoA hydrolase, mitochondrial OS=Homo sapiens OX=9606 GN=HIE	1.813472
40S ribosomal protein S20 OS=Homo sapiens OX=9606 GN=RPS20 PE=1 SV=1	10.08403
40S ribosomal protein S26 OS=Homo sapiens OX=9606 GN=RPS26 PE=1 SV=3	7.826087
60 kDa SS-A/Ro ribonucleoprotein OS=Homo sapiens OX=9606 GN=TROVE2 PE=1 SV=2	2.04461
60S ribosomal export protein NMD3 OS=Homo sapiens OX=9606 GN=NMD3 PE=1 SV=1	1.789264
60S ribosomal protein L36 OS=Homo sapiens OX=9606 GN=RPL36 PE=1 SV=3	9.52381

Actin-related protein 2/3 complex subunit 5 OS=Homo sapiens OX=9606 GN=ARPC5 PE=	7.94702
Adenylosuccinate lyase OS=Homo sapiens OX=9606 GN=ADSL PE=1 SV=2	1.859504
ADP-ribosylation factor 6 OS=Homo sapiens OX=9606 GN=ARF6 PE=1 SV=2	6.285714
ADP-ribosylation factor-like protein 1 OS=Homo sapiens OX=9606 GN=ARL1 PE=1 SV=1	6.077348
AFG3-like protein 2 OS=Homo sapiens OX=9606 GN=AFG3L2 PE=1 SV=2	4.015056
A-kinase anchor protein 2 OS=Homo sapiens OX=9606 GN=AKAP2 PE=1 SV=3	2.328289
Aldo-keto reductase family 1 member B15 OS=Homo sapiens OX=9606 GN=AKR1B15 PE	2.531646
Alpha-1,6-mannosyl-glycoprotein 2-beta-N-acetylglucosaminyltransferase OS=Homo sa	3.355705
Alpha-mannosidase 2 OS=Homo sapiens OX=9606 GN=MAN2A1 PE=1 SV=2	0.611888
Ameloblastin OS=Homo sapiens OX=9606 GN=AMBN PE=1 SV=1	2.46085
Armadillo-like helical domain-containing protein 3 OS=Homo sapiens OX=9606 GN=ARI	1.306241
Asparagine synthetase [glutamine-hydrolyzing] OS=Homo sapiens OX=9606 GN=ASNS PE	3.743316
ATPase family AAA domain-containing protein 1 OS=Homo sapiens OX=9606 GN=ATAD1	6.648199
ATP-binding cassette sub-family B member 7, mitochondrial OS=Homo sapiens OX=960	1.329787
ATR-interacting protein OS=Homo sapiens OX=9606 GN=ATRIP PE=1 SV=1	1.390645
Baculoviral IAP repeat-containing protein 6 OS=Homo sapiens OX=9606 GN=BIRC6 PE=1	0.205888
Beta-1,4-galactosyltransferase 7 OS=Homo sapiens OX=9606 GN=B4GALT7 PE=1 SV=1	2.446483
Brain-specific angiogenesis inhibitor 1-associated protein 2-like protein 1 OS=Homo sap	2.348337
BRCA1-associated ATM activator 1 OS=Homo sapiens OX=9606 GN=BRAT1 PE=1 SV=2	0.852619
Brefeldin A-inhibited guanine nucleotide-exchange protein 2 OS=Homo sapiens OX=960	0.840336
C2 domain-containing protein 5 OS=Homo sapiens OX=9606 GN=C2CD5 PE=1 SV=1	0.9
CAAX prenyl protease 1 homolog OS=Homo sapiens OX=9606 GN=ZMPSTE24 PE=1 SV=2	1.684211
Calcyclin-binding protein OS=Homo sapiens OX=9606 GN=CACYBP PE=1 SV=2	5.701754
Carbohydrate sulfotransferase 14 OS=Homo sapiens OX=9606 GN=CHST14 PE=1 SV=2	2.659574
Carnitine O-palmitoyltransferase 1, liver isoform OS=Homo sapiens OX=9606 GN=CPT1/	1.293661
Casein kinase I isoform alpha OS=Homo sapiens OX=9606 GN=CSNK1A1 PE=1 SV=2	2.670623
Caveolae-associated protein 3 OS=Homo sapiens OX=9606 GN=CAVIN3 PE=1 SV=3	4.214559
CCA tRNA nucleotidyltransferase 1, mitochondrial OS=Homo sapiens OX=9606 GN=TRN	3.456221
CD109 antigen OS=Homo sapiens OX=9606 GN=CD109 PE=1 SV=2	0.553633
Cdc42 effector protein 4 OS=Homo sapiens OX=9606 GN=CDC42EP4 PE=1 SV=1	3.932584
Cell division cycle protein 16 homolog OS=Homo sapiens OX=9606 GN=CDC16 PE=1 SV=	1.612903
Charged multivesicular body protein 1a OS=Homo sapiens OX=9606 GN=CHMP1A PE=1	4.591837
Cleavage and polyadenylation specificity factor subunit 7 OS=Homo sapiens OX=9606 G	1.698514
Complement C3 OS=Homo sapiens OX=9606 GN=C3 PE=1 SV=2	0.721587
Connective tissue growth factor OS=Homo sapiens OX=9606 GN=CTGF PE=1 SV=2	3.724928
Conserved oligomeric Golgi complex subunit 5 OS=Homo sapiens OX=9606 GN=COG5 P	1.549464
C-type lectin domain family 2 member B OS=Homo sapiens OX=9606 GN=CLEC2B PE=1 S	8.053691
Cytoplasmic dynein 1 light intermediate chain 1 OS=Homo sapiens OX=9606 GN=DYNC1	2.676864
Cytosolic non-specific dipeptidase OS=Homo sapiens OX=9606 GN=CNDP2 PE=1 SV=2	2.105263
Deaminated glutathione amidase OS=Homo sapiens OX=9606 GN=NIT1 PE=1 SV=2	2.140673
Dendrin OS=Homo sapiens OX=9606 GN=DDN PE=1 SV=3	1.828411
DnaJ homolog subfamily B member 1 OS=Homo sapiens OX=9606 GN=DNAJB1 PE=1 SV=	2.058824
Dolichol-phosphate mannosyltransferase subunit 1 OS=Homo sapiens OX=9606 GN=DP	4.230769
Dynactin subunit 4 OS=Homo sapiens OX=9606 GN=DCTN4 PE=1 SV=1	2.391304
E3 ubiquitin-protein ligase TRIM33 OS=Homo sapiens OX=9606 GN=TRIM33 PE=1 SV=3	0.976043
Echinoderm microtubule-associated protein-like 4 OS=Homo sapiens OX=9606 GN=EMI	2.446483
Endoplasmic reticulum metallopeptidase 1 OS=Homo sapiens OX=9606 GN=ERMP1 PE=	0.995575
Enhancer of mRNA-decapping protein 4 OS=Homo sapiens OX=9606 GN=EDC4 PE=1 SV=	0.571021
Ethylmalonyl-CoA decarboxylase OS=Homo sapiens OX=9606 GN=ECHDC1 PE=1 SV=2	3.257329
Eukaryotic translation initiation factor 3 subunit K OS=Homo sapiens OX=9606 GN=EIF3	5.045872
Eukaryotic translation initiation factor 6 OS=Homo sapiens OX=9606 GN=EIF6 PE=1 SV=	5.714286
Exocyst complex component 1 OS=Homo sapiens OX=9606 GN=EXOC1 PE=1 SV=4	1.006711

Fanconi anemia group D2 protein OS=Homo sapiens OX=9606 GN=FANCD2 PE=1 SV=2	0.551344
Fanconi anemia group I protein OS=Homo sapiens OX=9606 GN=FANCI PE=1 SV=4	0.753012
Fatty aldehyde dehydrogenase OS=Homo sapiens OX=9606 GN=ALDH3A2 PE=1 SV=1	4.536082
F-box/WD repeat-containing protein 2 OS=Homo sapiens OX=9606 GN=FBXW2 PE=1 SV	3.0837
Fibulin-1 OS=Homo sapiens OX=9606 GN=FBLN1 PE=1 SV=4	1.422475
Fragile X mental retardation syndrome-related protein 2 OS=Homo sapiens OX=9606 GN	1.188707
General transcription and DNA repair factor IIH helicase subunit XPB OS=Homo sapiens	1.023018
General transcription and DNA repair factor IIH helicase subunit XPD OS=Homo sapiens	0.921053
Glutamine-dependent NAD(+) synthetase OS=Homo sapiens OX=9606 GN=NADSYN1 PE=	0.991501
Glycine--tRNA ligase OS=Homo sapiens OX=9606 GN=GARS PE=1 SV=3	1.082544
Glycogen synthase kinase-3 beta OS=Homo sapiens OX=9606 GN=GSK3B PE=1 SV=2	5.714286
Golgi pH regulator B OS=Homo sapiens OX=9606 GN=GPR89B PE=1 SV=1	2.637363
Golgin subfamily A member 5 OS=Homo sapiens OX=9606 GN=GOLGA5 PE=1 SV=3	1.778386
Growth arrest-specific protein 6 OS=Homo sapiens OX=9606 GN=GAS6 PE=1 SV=2	1.664355
Growth-regulated alpha protein OS=Homo sapiens OX=9606 GN=CXCL1 PE=1 SV=1	11.21495
GTP-binding protein Di-Ras2 OS=Homo sapiens OX=9606 GN=DIRAS2 PE=1 SV=1	5.527638
HEAT repeat-containing protein 6 OS=Homo sapiens OX=9606 GN=HEATR6 PE=1 SV=1	1.016088
Helicase with zinc finger domain 2 OS=Homo sapiens OX=9606 GN=HELZ2 PE=1 SV=6	0.641752
Histone-lysine N-methyltransferase ASH1L OS=Homo sapiens OX=9606 GN=ASH1L PE=1	0.404176
Histone-lysine N-methyltransferase setd3 OS=Homo sapiens OX=9606 GN=SETD3 PE=1 S	2.020202
Huntingtin OS=Homo sapiens OX=9606 GN=HTT PE=1 SV=2	0.254615
Hyaluronan-binding protein 2 OS=Homo sapiens OX=9606 GN=HABP2 PE=1 SV=1	1.607143
Inactive serine protease 54 OS=Homo sapiens OX=9606 GN=PRSS54 PE=2 SV=3	2.78481
Inositol 1,4,5-trisphosphate receptor-interacting protein OS=Homo sapiens OX=9606 G	1.279707
Inositol monophosphatase 3 OS=Homo sapiens OX=9606 GN=IMPAD1 PE=1 SV=1	2.228412
Integral membrane protein 2C OS=Homo sapiens OX=9606 GN=ITM2C PE=1 SV=1	4.11985
Integrator complex subunit 10 OS=Homo sapiens OX=9606 GN=INTS10 PE=1 SV=2	1.126761
Integrator complex subunit 12 OS=Homo sapiens OX=9606 GN=INTS12 PE=1 SV=1	2.380952
Intraflagellar transport protein 56 OS=Homo sapiens OX=9606 GN=TTC26 PE=1 SV=1	2.34657
Isobutyryl-CoA dehydrogenase, mitochondrial OS=Homo sapiens OX=9606 GN=ACAD8 I	2.168675
Kinetochore protein Nuf2 OS=Homo sapiens OX=9606 GN=NUF2 PE=1 SV=2	2.801724
Kinetochore protein Spc24 OS=Homo sapiens OX=9606 GN=SPC24 PE=1 SV=2	3.553299
Lariat debranching enzyme OS=Homo sapiens OX=9606 GN=DBR1 PE=1 SV=2	2.022059
Latent-transforming growth factor beta-binding protein 1 OS=Homo sapiens OX=9606 (	0.929692
LETM1 domain-containing protein 1 OS=Homo sapiens OX=9606 GN=LETMD1 PE=1 SV=:	3.055556
LINE-1 type transposase domain-containing protein 1 OS=Homo sapiens OX=9606 GN=L	1.040462
Lipid droplet-associated hydrolase OS=Homo sapiens OX=9606 GN=LDAH PE=1 SV=1	2.461538
Lysophospholipid acyltransferase 5 OS=Homo sapiens OX=9606 GN=LPCAT3 PE=1 SV=1	2.669405
Maestro heat-like repeat-containing protein family member 1 OS=Homo sapiens OX=96(	0.548446
Metallothionein-1E OS=Homo sapiens OX=9606 GN=MT1E PE=1 SV=1	13.11475
Methionine synthase OS=Homo sapiens OX=9606 GN=MTR PE=1 SV=2	1.027668
Methylmalonate-semialdehyde dehydrogenase [acylating], mitochondrial OS=Homo sa|	3.738318
Mitochondrial carrier homolog 1 OS=Homo sapiens OX=9606 GN=MTCH1 PE=1 SV=1	2.570694
Mitochondrial import inner membrane translocase subunit TIM44 OS=Homo sapiens O:	2.654867
Mitochondrial inner membrane protein OXA1L OS=Homo sapiens OX=9606 GN=OXA1L	1.83908
Mitochondrial-processing peptidase subunit alpha OS=Homo sapiens OX=9606 GN=PMI	1.52381
Mitogen-activated protein kinase 13 OS=Homo sapiens OX=9606 GN=MAPK13 PE=1 SV=	5.205479
Mitotic spindle assembly checkpoint protein MAD2A OS=Homo sapiens OX=9606 GN=N	3.414634
Myotubularin-related protein 3 OS=Homo sapiens OX=9606 GN=MTMR3 PE=1 SV=3	0.584307
Myotubularin-related protein 5 OS=Homo sapiens OX=9606 GN=SBF1 PE=1 SV=4	0.535332
NACHT, LRR and PYD domains-containing protein 13 OS=Homo sapiens OX=9606 GN=NI	0.671141
N-alpha-acetyltransferase 16, NatA auxiliary subunit OS=Homo sapiens OX=9606 GN=N/	0.810185

N-alpha-acetyltransferase 30 OS=Homo sapiens OX=9606 GN=NAA30 PE=1 SV=1	3.314917
Neurofibromin OS=Homo sapiens OX=9606 GN=NF1 PE=1 SV=2	0.317013
Nibrin OS=Homo sapiens OX=9606 GN=NBN PE=1 SV=1	3.580902
Nuclear pore complex protein Nup214 OS=Homo sapiens OX=9606 GN=NUP214 PE=1 S	0.574163
Nuclear receptor subfamily 0 group B member 1 OS=Homo sapiens OX=9606 GN=NR0B1	3.404255
Nucleolar GTP-binding protein 1 OS=Homo sapiens OX=9606 GN=GTPBP4 PE=1 SV=3	2.050473
Obg-like ATPase 1 OS=Homo sapiens OX=9606 GN=OLA1 PE=1 SV=2	2.777778
PCI domain-containing protein 2 OS=Homo sapiens OX=9606 GN=PCID2 PE=1 SV=2	4.010025
Phosphorylase b kinase regulatory subunit alpha, liver isoform OS=Homo sapiens OX=96	1.133603
Pleckstrin homology-like domain family A member 2 OS=Homo sapiens OX=9606 GN=PI	5.921053
Pregnancy zone protein OS=Homo sapiens OX=9606 GN=PZP PE=1 SV=4	0.607287
Presequence protease, mitochondrial OS=Homo sapiens OX=9606 GN=PITRM1 PE=1 SV=	2.603664
Probable cytosolic iron-sulfur protein assembly protein CIAO1 OS=Homo sapiens OX=96	3.539823
Probable global transcription activator SNF2L1 OS=Homo sapiens OX=9606 GN=SMARC	0.664137
Probable leucine--tRNA ligase, mitochondrial OS=Homo sapiens OX=9606 GN=LARS2 PE	2.325581
Prolactin regulatory element-binding protein OS=Homo sapiens OX=9606 GN=PREB PE=	2.877698
Proline-serine-threonine phosphatase-interacting protein 2 OS=Homo sapiens OX=9606	2.694611
Prolyl 3-hydroxylase 2 OS=Homo sapiens OX=9606 GN=P3H2 PE=1 SV=1	1.129944
Prostaglandin E synthase 3 OS=Homo sapiens OX=9606 GN=PTGES3 PE=1 SV=1	8.125
Proteasome inhibitor PI31 subunit OS=Homo sapiens OX=9606 GN=PSMF1 PE=1 SV=2	4.428044
Proteasome subunit beta type-3 OS=Homo sapiens OX=9606 GN=PSMB3 PE=1 SV=2	3.414634
Protein ABHD14B OS=Homo sapiens OX=9606 GN=ABHD14B PE=1 SV=1	6.190476
Protein CASP OS=Homo sapiens OX=9606 GN=CUX1 PE=1 SV=2	1.327434
Protein NDRG3 OS=Homo sapiens OX=9606 GN=NDRG3 PE=1 SV=2	2.666667
Protein NEDD1 OS=Homo sapiens OX=9606 GN=NEDD1 PE=1 SV=1	1.666667
Protein quaking OS=Homo sapiens OX=9606 GN=QKI PE=1 SV=1	2.932551
Protein RER1 OS=Homo sapiens OX=9606 GN=RER1 PE=1 SV=1	4.081633
Prothymosin alpha OS=Homo sapiens OX=9606 GN=PTMA PE=1 SV=2	12.61261
Pyridoxal-dependent decarboxylase domain-containing protein 1 OS=Homo sapiens OX	1.649746
Pyrroline-5-carboxylate reductase 3 OS=Homo sapiens OX=9606 GN=PYCR3 PE=1 SV=3	5.109489
Raftlin OS=Homo sapiens OX=9606 GN=RFTN1 PE=1 SV=4	3.460208
Ral GTPase-activating protein subunit beta OS=Homo sapiens OX=9606 GN=RALGAPB PI	0.803213
Rapamycin-insensitive companion of mTOR OS=Homo sapiens OX=9606 GN=RICTOR PE=	0.644028
Ras GTPase-activating protein 1 OS=Homo sapiens OX=9606 GN=RASA1 PE=1 SV=1	1.337154
Receptor tyrosine-protein kinase erbB-4 OS=Homo sapiens OX=9606 GN=ERBB4 PE=1 SV	0.611621
Regulator of nonsense transcripts 1 OS=Homo sapiens OX=9606 GN=UPF1 PE=1 SV=2	0.974314
RelA-associated inhibitor OS=Homo sapiens OX=9606 GN=PPP1R13L PE=1 SV=4	1.811594
Reticulon-4 OS=Homo sapiens OX=9606 GN=RTN4 PE=1 SV=2	1.090604
Rho guanine nucleotide exchange factor 28 OS=Homo sapiens OX=9606 GN=ARHGEF28	0.645161
Rho guanine nucleotide exchange factor 7 OS=Homo sapiens OX=9606 GN=ARHGEF7 PE	1.24533
Ribonucleoside-diphosphate reductase subunit M2 B OS=Homo sapiens OX=9606 GN=R	4.558405
RIMS-binding protein 3C OS=Homo sapiens OX=9606 GN=RIMBP3C PE=1 SV=3	0.42709
RNA cytidine acetyltransferase OS=Homo sapiens OX=9606 GN=NAT10 PE=1 SV=2	2.439024
RNA polymerase II-associated protein 1 OS=Homo sapiens OX=9606 GN=RPAP1 PE=1 SV=	0.502513
RUN and FYVE domain-containing protein 2 OS=Homo sapiens OX=9606 GN=RUFY2 PE=1	2.145215
Septin-4 OS=Homo sapiens OX=9606 GN=SEPT4 PE=1 SV=1	1.882845
Serine/arginine repetitive matrix protein 2 OS=Homo sapiens OX=9606 GN=SRRM2 PE=1	0.508721
Serine/arginine-rich splicing factor 4 OS=Homo sapiens OX=9606 GN=SRSF4 PE=1 SV=2	1.417004
Serine/arginine-rich splicing factor 7 OS=Homo sapiens OX=9606 GN=SRSF7 PE=1 SV=1	3.781513
Serine/threonine-protein kinase 11-interacting protein OS=Homo sapiens OX=9606 GN=	1.011029
Serine/threonine-protein kinase LMTK2 OS=Homo sapiens OX=9606 GN=LMTK2 PE=1 SV	0.598802
Serine/threonine-protein kinase Nek2 OS=Homo sapiens OX=9606 GN=NEK2 PE=1 SV=1	2.47191

Serine/threonine-protein kinase Nek7 OS=Homo sapiens OX=9606 GN=NEK7 PE=1 SV=1	4.304636
Serine/threonine-protein phosphatase 6 regulatory subunit 1 OS=Homo sapiens OX=96(	1.021566
Serine/threonine-protein phosphatase PGAM5, mitochondrial OS=Homo sapiens OX=9(	3.460208
Serum paraoxonase/arylesterase 2 OS=Homo sapiens OX=9606 GN=PON2 PE=1 SV=4	4.519774
Serum response factor-binding protein 1 OS=Homo sapiens OX=9606 GN=SRFBP1 PE=1 !	3.263403
Short/branched chain specific acyl-CoA dehydrogenase, mitochondrial OS=Homo sapie	2.083333
Signal transducer and activator of transcription 2 OS=Homo sapiens OX=9606 GN=STAT:	1.292597
Solute carrier family 25 member 46 OS=Homo sapiens OX=9606 GN=SLC25A46 PE=1 SV:	2.392344
Sorting nexin-1 OS=Homo sapiens OX=9606 GN=SNX1 PE=1 SV=3	2.10728
Sorting nexin-7 OS=Homo sapiens OX=9606 GN=SNX7 PE=1 SV=1	2.842377
Sperm-specific antigen 2 OS=Homo sapiens OX=9606 GN=SSFA2 PE=1 SV=3	1.032566
Sphingomyelin phosphodiesterase 4 OS=Homo sapiens OX=9606 GN=SMPD4 PE=1 SV=3	3.117783
Splicing factor U2AF 65 kDa subunit OS=Homo sapiens OX=9606 GN=U2AF2 PE=1 SV=4	1.684211
Stress-induced-phosphoprotein 1 OS=Homo sapiens OX=9606 GN=STIP1 PE=1 SV=1	2.394107
SWI/SNF-related matrix-associated actin-dependent regulator of chromatin subfamily A	0.974659
TBC1 domain family member 9B OS=Homo sapiens OX=9606 GN=TBC1D9B PE=1 SV=3	2
Tensin-1 OS=Homo sapiens OX=9606 GN=TNS1 PE=1 SV=2	0.74928
Terminal uridylyltransferase 7 OS=Homo sapiens OX=9606 GN=TUT7 PE=1 SV=1	0.802676
THO complex subunit 3 OS=Homo sapiens OX=9606 GN=THOC3 PE=1 SV=1	2.564103
T-lymphoma invasion and metastasis-inducing protein 2 OS=Homo sapiens OX=9606 GI	0.764256
Transcription elongation regulator 1 OS=Homo sapiens OX=9606 GN=TCERG1 PE=1 SV=:	0.819672
Translin OS=Homo sapiens OX=9606 GN=TSN PE=1 SV=1	6.578947
Translocation protein SEC62 OS=Homo sapiens OX=9606 GN=SEC62 PE=1 SV=1	2.255639
Translocon-associated protein subunit alpha OS=Homo sapiens OX=9606 GN=SSR1 PE=1	2.797203
Transmembrane and TPR repeat-containing protein 3 OS=Homo sapiens OX=9606 GN=T	0.983607
Transmembrane emp24 domain-containing protein 4 OS=Homo sapiens OX=9606 GN=1	3.964758
Transmembrane emp24 domain-containing protein 9 OS=Homo sapiens OX=9606 GN=1	4.680851
Transmembrane protein 87A OS=Homo sapiens OX=9606 GN=TMEM87A PE=1 SV=3	1.261261
Tyrosine-protein kinase JAK3 OS=Homo sapiens OX=9606 GN=JAK3 PE=1 SV=2	0.711744
Tyrosine--tRNA ligase, cytoplasmic OS=Homo sapiens OX=9606 GN=YARS PE=1 SV=4	1.704545
Ubiquitin carboxyl-terminal hydrolase 14 OS=Homo sapiens OX=9606 GN=USP14 PE=1	1.417004
Ubiquitin carboxyl-terminal hydrolase 25 OS=Homo sapiens OX=9606 GN=USP25 PE=1	1.232227
Ubiquitin conjugation factor E4 A OS=Homo sapiens OX=9606 GN=UBE4A PE=1 SV=2	1.125704
Ubiquitin-conjugating enzyme E2 K OS=Homo sapiens OX=9606 GN=UBE2K PE=1 SV=3	5.5
Ubiquitin-like protein ISG15 OS=Homo sapiens OX=9606 GN=ISG15 PE=1 SV=5	5.454545
Ubiquitin-protein ligase E3C OS=Homo sapiens OX=9606 GN=UBE3C PE=1 SV=3	1.108033
UDP-N-acetylhexosamine pyrophosphorylase-like protein 1 OS=Homo sapiens OX=9606	1.972387
UPF0160 protein MYG1, mitochondrial OS=Homo sapiens OX=9606 GN=C12orf10 PE=1	2.659574
Uridine-cytidine kinase 2 OS=Homo sapiens OX=9606 GN=UCK2 PE=1 SV=1	6.896552
Vacuolar protein sorting-associated protein 4A OS=Homo sapiens OX=9606 GN=VPS4A I	2.517162
Vasodilator-stimulated phosphoprotein OS=Homo sapiens OX=9606 GN=VASP PE=1 SV=	2.368421
Volume-regulated anion channel subunit LRRC8C OS=Homo sapiens OX=9606 GN=LRRC	0.996264
WASH complex subunit 3 OS=Homo sapiens OX=9606 GN=WASHC3 PE=1 SV=1	3.608247
WASH complex subunit 4 OS=Homo sapiens OX=9606 GN=WASHC4 PE=1 SV=2	0.682012
WD repeat-containing protein 3 OS=Homo sapiens OX=9606 GN=WDR3 PE=1 SV=1	0.848356
WD repeat-containing protein 43 OS=Homo sapiens OX=9606 GN=WDR43 PE=1 SV=3	1.033973
YTH domain-containing family protein 3 OS=Homo sapiens OX=9606 GN=YTHDF3 PE=1 !	1.196581
Zinc finger CCCH domain-containing protein 11A OS=Homo sapiens OX=9606 GN=ZC3H	1.851852
Zinc finger CCHC domain-containing protein 8 OS=Homo sapiens OX=9606 GN=ZCCHC8	1.980198
Zinc finger MYM-type protein 3 OS=Homo sapiens OX=9606 GN=ZMYM3 PE=1 SV=2	1.094891
Zinc finger protein 622 OS=Homo sapiens OX=9606 GN=ZNF622 PE=1 SV=1	1.886792
Zinc finger protein ubi-d4 OS=Homo sapiens OX=9606 GN=DPF2 PE=1 SV=2	3.836317

Zinc finger SWIM domain-containing protein 8 OS=Homo sapiens OX=9606 GN=ZSWIM8	0.653239
Zinc transporter ZIP11 OS=Homo sapiens OX=9606 GN=SLC39A11 PE=2 SV=3	4.97076

# Peptides	# PSMs	# Unique Pe	# AAs	MW [kDa]	calc. pI	Score Mascot
8	8	8	913	105.278	5.68	256.8837025
9	15	9	411	46.757	5.26	248.3529406
6	7	1	641	70.331	6.02	192.3028571
4	5	3	864	97.347	7.17	176.38
3	3	3	594	66.487	5.59	162.32
3	6	2	365	40.82	6.54	150.1676689
4	4	4	928	103.07	4.78	146.77
5	9	1	463	50.438	9.03	138.9929511
1	2	1	306	32.729	5.06	136.0003258
1	1	1	1443	160.782	6.4	126.22
3	4	3	319	33.34	7.61	124.12
1	2	1	770	88.011	6.3	112.1
1	2	1	539	58.179	7.64	108.49
4	4	2	393	43.411	6.62	104.91
3	3	3	593	65.22	9.7	104.0633333
4	4	2	365	38.556	6.79	103.89
1	1	1	953	105.247	5.71	90.3
4	4	4	1478	164.876	5.22	86.01416763
3	4	3	2549	288.707	7.17	85.62333333
2	2	2	669	73.414	7.77	84.28379381
5	5	5	808	89.575	6.3	84.01
1	3	1	127	14.159	10.32	83.37401457
8	8	8	2671	303.912	6.48	80.62316161
3	4	2	648	73.005	9.2	79.93
1	1	1	157	18.603	5.47	79.69
6	6	5	949	104.57	5.06	78.62
1	1	1	487	56.216	6.25	75.61
2	3	2	759	87.624	8.13	75.44
2	3	1	606	67.542	9.01	75.37
1	1	1	980	108.909	7.31	73.5
1	1	1	2109	238.725	7.3	73.1
1	1	1	349	40.26	5.57	70.88
3	3	3	475	54.454	8.7	69.58
3	4	3	1153	130.076	8.48	68.85333333
3	3	3	279	32.171	7.55	68.57
1	1	1	1992	220.296	8.48	68.46
2	2	2	917	98.998	9.07	68.01
1	1	1	724	79.338	6.55	67.43
3	3	2	359	42.115	5.68	67.13834151
4	4	3	489	54.331	6.83	66.91392193
4	4	4	2620	294.178	6.14	66.02745402
1	2	1	456	50.683	9.42	65.82
1	1	1	253	28.143	9.54	65.32
4	4	4	912	102.371	5.66	65.01666667
4	4	2	346	38.901	7.87	63.81848109
4	5	2	1241	137.833	6.6	63.72333333
1	1	1	963	107.428	7.71	62.88
6	8	5	406	45.597	7.55	62.16666667
1	1	1	125	14.454	10.54	61.86
2	2	2	1006	111.581	6.68	61.48
3	3	3	1845	204.16	7.12	61.34

2	2	2	269	30.728	4.83	61.04
1	1	1	351	40.597	8.78	60.51
3	4	3	318	34.812	6.98	60.34986473
2	2	2	350	39.285	9.17	60.18
1	1	1	482	55.83	6.48	60.12
2	2	2	1636	178.861	6.92	59.82
2	2	2	301	34.213	7.59	59.64
1	1	1	216	23.728	9.41	58.55
1	1	1	741	83.489	5.69	58.07
1	1	1	692	76.061	5.15	57.45
1	1	1	1146	127.513	7.64	56.76
2	2	2	430	47.976	9.92	56.5
1	1	1	1024	114.636	9.22	56.12
1	1	1	215	24.578	6.92	55.95
3	3	3	891	100.99	8.22	55.71243666
1	1	1	1105	124.633	5.01	55.45
1	1	1	93	10.136	9.79	55.38
1	1	1	228	25.677	7.25	54.92
1	1	1	802	85.074	6.79	54.53
3	3	3	1647	184.53	7.88	54.3
2	2	2	353	39.622	8.37	54.05
3	4	3	221	25.461	11.85	53.64666667
3	3	3	559	63.239	8.4	53.51
1	1	1	346	39.225	5.5	53.48
1	1	1	711	82.201	7.61	53.42
3	3	3	418	46.909	6.93	53.39968559
1	1	1	7570	860.127	5.25	52.73
1	2	1	821	92.831	5.41	52.39
1	1	1	335	36.403	6.35	51.64
1	1	1	440	47.657	5.22	51.42
2	2	1	420	46.409	9.57	51.38
3	3	3	2785	318.182	7.39	51.09666667
1	1	1	394	42.808	5.82	49.86
2	2	2	319	35.255	8.76	49.82
1	1	1	299	33.374	5.25	49.62
1	1	1	424	44.357	8.56	49.52
1	1	1	847	94.075	6.23	49.44
1	1	1	374	41.714	4.92	49.25
1	1	1	875	94.985	7.97	48.8
1	1	1	816	88.331	5.88	47.96
1	1	1	964	111.104	6.2	47.76
3	3	3	340	38.472	7.2	47.55542488
1	1	1	517	57.362	6.76	47.54
1	1	1	702	79.272	9.13	47.53
1	1	1	419	49.481	5.69	47.44
1	1	1	211	23.61	8.65	46.87
1	1	1	524	58.006	5.96	46.77
1	1	1	348	38.608	9.48	46.4
2	2	2	534	59.113	6.02	45.84
1	1	1	155	17.587	9.39	45.62
3	3	3	1384	156.167	7.05	45.56954773
1	1	1	360	41.254	7.9	45.44

1	1	1	904	102.274	11.84	45.41
3	5	3	428	48.96	5.67	45.13182403
4	4	4	372	42.75	8.79	44.89333333
1	1	1	427	47.19	5.34	44.71
1	1	1	1498	168.783	7.5	44.49
1	1	1	212	24.526	8.62	44.45
1	1	1	314	34.413	9.16	44.41
1	2	1	483	53.185	8.47	44.08
1	1	1	337	37.974	9.19	43.72
2	2	2	176	20.108	10.13	43.48479304
2	2	2	764	85.926	5.83	43.22
2	2	2	519	56.131	7.93	43.12
1	1	1	711	79.137	8.38	43.07
1	1	1	881	97.355	9.33	42.91
1	1	1	579	63.666	8.87	42.75
1	1	1	725	80.422	7.78	42.47
1	1	1	284	33.289	9.31	42.4
1	1	1	468	50.985	6.93	42.32
1	1	1	163	17.652	5.19	42.25
1	1	1	492	54.177	5.33	42.22
1	1	1	735	83.33	6.79	42.2
1	1	1	490	55.95	5.2	42.14
1	1	1	538	60.184	5.01	42.13
2	2	2	417	48.112	4.44	41.61
1	1	1	963	108.102	6.44	41.57
1	2	1	881	95.377	7.62	40.81
1	2	1	355	37.953	5.26	40.26
1	1	1	1113	124.889	7.02	40.03
4	4	3	614	69.457	8.03	40.02
2	2	2	775	82.923	6.28	40.0178866
3	3	3	894	98.274	5.39	39.97
1	1	1	227	25.55	9.16	39.59
3	3	3	2863	318.906	5.6	39.49
1	1	1	319	34.061	9.04	39.48
1	1	1	371	41.909	9.35	39.46
1	2	1	298	33.79	6.44	39.42
2	2	2	549	60.87	7.33	39.12
2	2	2	218	24.564	6.68	39.11
1	1	1	745	82.791	7.37	39.08
1	1	1	562	62.876	7.8	39.01
3	3	3	476	52.231	8.06	38.92184916
3	3	2	624	70.788	6.86	38.01
1	2	1	339	38.274	8.32	37.88
2	2	2	2067	217.092	6.39	37.52
1	1	1	1224	136.598	6.92	37.01
1	1	1	1766	200.423	6.25	36.64
1	1	1	610	66.777	8.28	36.46
3	3	2	419	48.013	5.92	36.43099135
2	2	2	412	45.717	6.48	36.42
1	1	1	1179	134.464	6.9	36.24
1	1	1	218	24.473	5.94	36.2
2	4	1	893	97.843	5.66	36.06

1	2	1	926	102.066	6.64	35.84
2	3	2	311	33.991	9.89	34.63163139
3	3	3	1070	118.317	7.09	34.47
2	2	1	594	64.016	8.32	34.32777943
1	1	1	1130	119.624	4.34	34.06
3	3	2	419	48.132	6.68	33.94235777
5	5	5	617	68.26	5.52	33.74331981
1	1	1	1475	153.843	8.73	33.39
1	1	1	466	51.364	4.97	33.26
1	1	1	461	52.807	9.33	32.96
1	1	1	180	19.595	6.02	32.56
1	1	1	1887	204.983	6.81	32.5
2	2	2	1454	160.504	8.73	32.26
1	1	1	1179	127.044	6.98	32.13
1	1	1	467	54.664	6.95	32.09
1	1	1	980	109.973	6.21	31.99
4	4	4	851	91.118	5.63	31.96
1	1	1	647	71.904	6.99	31.93
1	1	1	780	90.897	7.94	31.83
1	1	1	330	37.167	6.29	31.78
1	1	1	309	35.552	5.43	31.69
1	1	1	214	22.073	8.7	31.57
1	1	1	195	20.999	9.54	31.53
1	1	1	538	62.189	8.56	30.57
1	1	1	715	77.496	9.01	30.49
1	1	1	841	95.931	5.11	30.43
1	2	1	788	85.186	4.3	30.16
1	1	1	1292	141.205	5.12	30.12
1	1	1	248	27.06	4.51	30.12
1	1	1	334	36.403	7.36	30.1
1	1	1	427	47.326	7.49	30.04
1	1	1	269	30.374	7.78	30.04
1	1	1	1849	198.876	8.31	29.91
1	1	1	631	73.152	8.15	29.86
1	1	1	314	34.574	8.24	29.86
1	2	1	176	20.749	10.71	29.7
1	1	1	1151	130.056	5.05	29.69
1	1	1	148	17.249	9.6	29.53
1	1	1	671	74.272	7.49	28.98
1	1	1	1120	125.35	5.91	28.86
2	2	2	645	73.516	8.02	28.84
2	2	2	725	81.747	5.49	28.52
2	2	2	736	83.341	5.14	28.51
1	1	1	429	49.944	6.6	28.24
1	1	1	1249	135.54	5.38	28.11
2	2	2	308	36.011	9.45	28.02921075
2	2	2	1222	134.236	7.2	28.02
1	2	1	639	68.286	8.98	27.8
3	3	3	737	82.538	5.1	27.64
1	1	1	981	110.454	5.55	27.38
1	1	1	592	61.793	8.02	27.36
1	1	1	812	89.043	8.44	27.29

1	1	1	483	55.847	6.48	27.22
2	2	1	286	32.902	5.69	27.11
2	2	2	570	61.924	6.49	27.09
2	2	2	491	53.803	8.44	26.74
1	1	1	541	62.212	7.36	26.66
3	3	2	298	33.908	8.68	26.64
1	1	1	805	88.284	8.32	26.56
1	1	1	1162	129.508	8.37	26.53
2	2	2	3056	350.462	6.81	26.5
1	1	1	69	7.928	9.35	26.48
3	3	2	330	37.488	6.33	26.32
2	2	2	1204	132.87	8.13	26.3
1	1	1	324	36.922	9.28	26.27
1	1	1	491	55.501	6.74	26.12
1	1	1	875	96.562	6.38	25.99
1	1	1	1219	133.547	5.2	25.93
3	3	3	913	101.304	5.45	25.89
1	1	1	5596	632.42	5.68	25.65
1	1	1	381	43.638	9.77	25.63
1	1	1	1577	177.236	5.39	25.35
1	1	1	135	15.85	11.33	25.33
2	2	2	713	80.589	7.05	25.3
2	2	2	464	52.484	7.23	25.25
3	3	3	2440	270.044	7.11	25.16
1	1	1	470	52.086	5.1	25
1	1	1	709	79.695	6.34	24.95
1	1	1	2382	250.64	6.34	24.88
1	1	1	570	62.569	9.61	24.74
1	1	1	229	25.711	8.82	24.65
1	1	1	334	36.565	8.27	24.63
1	1	1	330	37.612	6.29	24.39
1	1	1	1755	200.408	6.24	24.32
1	1	1	745	81.693	6.14	24.21
1	1	1	505	58.217	6.67	24.18
1	1	1	391	40.27	7.15	24.16
1	1	1	509	58.769	7.91	24.11
2	2	2	1649	186.794	6.28	24.06
2	2	2	4579	520.795	7.05	24.06
1	1	1	839	94.387	6.67	24.05
1	1	1	417	46.915	8.09	24.02
1	1	1	1943	213.983	7.06	23.97
2	2	2	968	112.065	6.95	23.96
1	1	1	1108	126.982	8.92	23.74
1	1	1	901	104.138	6.55	23.71
1	1	1	245	26.366	6.52	23.69
3	3	3	429	47.121	9.14	23.58
1	1	1	508	57.081	8.44	23.58
1	1	1	261	29.797	5.55	23.52
1	1	1	194	21.621	8.63	23.38
1	1	1	875	100.623	5.22	23.28
1	1	1	344	38.938	9.01	23.26
2	2	2	889	101.132	6.96	22.91

1	1	1	271	28.807	5.83	22.88
1	1	1	855	99.946	6.23	22.78
1	1	1	158	18.031	6.74	22.44
2	2	1	1173	130.7	7.08	22.43
3	3	3	626	72.155	7.14	22.38
1	1	1	1706	193.985	7.21	22.36
2	2	2	863	98.201	6.46	22.22
2	2	2	578	65.658	8.38	21.87
2	2	2	285	29.625	6.01	21.71
1	1	1	1538	169.346	9.03	21.58
1	1	1	826	92.441	5.96	21.52
2	2	2	975	107.317	7.21	21.48
1	1	1	709	79.614	5.41	21.42
2	2	2	223	25.252	8.4	21.3645098
1	1	1	219	24.742	5.02	21.27
1	2	1	281	33.334	9.26	21.23
1	1	1	744	82.257	7.78	21.12
1	1	1	611	69.818	8.68	21.1
2	2	2	351	40.303	5	20.92
1	1	1	608	68.077	6.38	20.88
1	1	1	447	51.659	6.2	20.87
1	1	1	672	75.642	4.75	20.76
1	1	1	282	31.259	9.83	20.64
1	1	1	410	44.932	5.63	20.56
1	1	1	1116	128.008	5.97	20.54
1	1	1	301	32.922	9.41	20.52
2	2	2	558	62.568	7.83	20.41
1	1	1	582	64.8	5.29	20.4
1	1	1	1912	214.625	6.57	20.29
1	1	1	602	69.947	8.13	20.2
2	2	2	975	111.862	5.82	20.07
3	3	3	379	43.108	6.74	19.92666667
1	1	1	672	76.7	7.05	19.88
2	2	2	785	87.062	6.55	19.55
1	1	1	218	25.634	9.98	19.5
1	1	1	746	83.577	6.19	19.44
2	2	2	603	68.212	8.91	19.34
1	1	1	365	40.998	5.16	19.25
4	4	4	1012	113.719	7.2	19.22
1	1	1	435	45.774	7.2	19.08
1	1	1	452	51.386	8.07	19.04
2	2	2	617	70.54	6.71	18.88
1	1	1	1258	138.513	6.54	18.68
2	2	2	601	68.31	5.73	18.6
2	2	1	825	92.507	7.96	18.42
1	1	1	528	56.594	6.64	18.1
1	1	1	191	21.295	8.12	18.07
1	1	1	426	45.992	8.53	18.02
2	2	2	1059	112.142	7.3	18.01
1	1	1	172	20.763	8.48	18.01
1	1	1	2539	284.395	6.39	17.8
1	1	1	275	32.426	5.77	17.78

1	1	1	430	48.178	5.05	17.76
1	1	1	508	56.905	8.15	17.75
1	1	1	209	23.866	9.35	17.68
1	1	1	672	74.438	5.12	17.64
1	1	1	3013	339.383	5.58	17.48
1	1	1	631	70.693	7.42	17.43
1	1	1	993	109.082	8.97	17.29
1	1	1	788	87	5.24	17.14
1	1	1	1132	119.334	5.6	17.12
1	1	1	205	24.197	10.39	16.9
1	1	1	323	35.673	5.16	16.82
1	1	1	565	65.34	8.91	16.79
1	1	1	258	29.045	4.83	16.72
1	1	1	1050	116.91	6.87	16.68
1	1	1	613	64.994	8	16.12
2	2	2	280	31.609	8.76	15.94
2	2	2	431	48.327	6.58	15.91
2	3	1	535	60.849	6.57	15.87
1	1	1	833	95.308	4.94	15.82
1	1	1	699	78.718	6.73	15.72
1	1	1	539	59.472	5.59	15.59
1	1	1	1749	195.917	6.73	15.56
2	3	2	1087	121.093	8.4	15.48
1	1	1	207	23.156	8.1	15.44
2	2	2	1817	197.457	6.4	15.44
1	1	1	227	26.136	8.28	15.43
2	2	2	897	100.921	6.9	15.4
2	2	2	347	39.293	7.43	15.37
1	1	1	1072	119.575	6.57	15.37
2	2	2	296	33.228	9.26	15.15
1	1	1	583	66.208	8.18	15.07
1	1	1	1202	135.695	6.29	14.85
1	1	1	561	62.007	5.02	14.85
1	1	1	481	54.293	6.23	14.69
1	1	1	283	31.815	9.92	14.61
1	1	1	246	27.406	9.5	14.58
1	1	1	795	88.179	8.69	14.57
2	2	2	1508	168.483	6.62	14.54
1	1	1	1099	124.425	6.68	14.47
1	1	1	515	56.071	6.32	14.26
2	2	2	219	24.96	7.44	14.22
1	1	1	419	47.504	8.5	13.75
1	1	1	279	31.492	8.35	13.74
1	1	1	1001	115.997	7.65	13.65
1	1	1	650	73.908	4.88	13.54
1	1	1	308	33.371	5.69	0
1	1	1	386	43.454	8.19	0
1	1	1	119	13.364	9.94	0
1	1	1	115	13.007	11	0
1	1	1	538	60.631	8.03	0
1	1	1	503	57.566	7.14	0
1	1	1	105	12.246	11.59	0

1	1	1	151	16.31	5.67	0
1	1	1	484	54.854	7.11	0
1	1	1	175	20.069	8.95	0
1	1	1	181	20.404	5.72	0
3	3	3	797	88.528	8.66	0
1	1	1	859	94.603	5.11	0
1	1	1	316	36.514	6.7	0
2	2	2	447	51.517	8.76	0
1	1	1	1144	131.057	7.58	0
1	1	1	447	48.252	4.92	0
1	2	1	689	78.659	6.6	0
2	2	2	561	64.329	6.86	0
2	2	2	361	40.718	6.9	0
1	1	1	752	82.589	9.33	0
1	1	1	791	85.783	6.32	0
1	1	1	4857	529.919	6.05	0
1	1	1	327	37.382	8.98	0
1	1	1	511	56.847	8.68	0
1	1	1	821	88.063	5.27	0
1	1	1	1785	201.909	6.33	0
1	1	1	1000	110.377	5.69	0
1	1	1	475	54.778	7.49	0
1	1	1	228	26.194	8.25	0
1	1	1	376	42.969	9.48	0
1	1	1	773	88.311	8.65	0
1	1	1	337	38.89	9.57	0
1	1	1	261	27.685	6.43	0
2	2	2	434	50.096	8.1	0
1	1	1	1445	161.587	5.85	0
1	1	1	356	37.957	5.19	0
1	1	1	620	71.609	5.85	0
1	1	1	196	21.689	8.06	0
1	1	1	471	52.018	8	0
1	1	1	1663	187.03	6.4	0
1	1	1	349	38.065	8	0
1	1	1	839	92.685	6.6	0
1	1	1	149	17.295	8.75	0
1	1	1	523	56.544	6.42	0
1	1	1	475	52.845	5.97	0
1	1	1	327	35.873	7.74	0
1	1	1	711	75.95	10.17	0
1	1	1	340	38.02	8.63	0
1	1	1	260	29.616	9.57	0
1	1	1	460	52.304	7.34	0
1	1	1	1127	122.456	6.67	0
2	2	2	981	108.848	6.4	0
1	1	1	904	100.167	7.52	0
1	1	1	1401	151.567	5.86	0
1	1	1	307	33.677	8.21	0
1	1	1	218	25.043	4.93	0
1	1	1	245	26.582	4.68	0
1	1	1	894	101.917	6.61	0

1	1	1	1451	164.025	5.88	0
1	1	1	1328	149.229	6.74	0
2	2	2	485	54.813	7.88	0
1	1	1	454	51.479	6.58	0
1	1	1	703	77.162	5.22	0
1	1	1	673	74.178	6.23	0
1	1	1	782	89.221	7.23	0
1	1	1	760	86.854	7.15	0
1	1	1	706	79.234	6.44	0
1	1	1	739	83.113	7.03	0
1	1	1	420	46.715	8.78	0
1	1	1	455	52.882	9.28	0
1	1	1	731	82.974	5.83	0
1	1	1	721	79.625	6.21	0
1	1	1	107	11.294	10.43	0
1	1	1	199	22.471	8.76	0
1	1	1	1181	128.699	7.03	0
1	1	1	2649	294.465	7.49	0
1	1	1	2969	332.582	9.39	0
1	1	1	594	67.215	5.96	0
1	1	1	3142	347.383	6.2	0
1	1	1	560	62.63	6.54	0
1	1	1	395	43.804	6.7	0
1	1	1	547	62.019	5.88	0
1	1	1	359	38.657	6.86	0
1	1	1	267	30.204	8	0
1	1	1	710	82.183	7.44	0
1	1	1	462	48.777	9.69	0
1	1	1	554	64.136	6.93	0
1	1	1	415	45.04	7.85	0
1	1	1	464	54.269	8.27	0
1	1	1	197	22.464	4.7	0
1	1	1	544	61.516	5.47	0
1	1	1	1721	186.673	5.96	0
1	1	1	360	41.763	10.32	0
1	1	1	865	98.789	4.93	0
1	1	1	325	37.294	6.54	0
1	1	1	487	55.998	8.69	0
1	1	1	1641	181.134	6.89	0
1	1	1	61	6.009	7.96	0
1	1	1	1265	140.437	5.58	0
1	1	1	535	57.803	8.5	0
1	1	1	389	41.517	9.32	0
1	1	1	452	51.323	8.32	0
1	1	1	435	48.516	9.45	0
1	1	1	525	58.216	6.92	0
2	2	2	365	42.063	8.38	0
1	1	1	205	23.495	5.08	0
1	1	1	1198	133.534	5.8	0
1	1	1	1868	208.312	6.9	0
1	1	1	1043	118.807	5.66	0
1	1	1	864	101.397	7.87	0

1	1	1	362	39.295	5.52	0
1	1	1	2839	319.168	7.39	0
2	2	2	754	84.906	6.9	0
1	1	1	2090	213.488	7.47	0
1	1	1	470	51.684	8.07	0
1	1	1	634	73.918	9.5	0
1	2	1	396	44.715	7.81	0
1	1	1	399	46	8.53	0
1	1	1	1235	138.32	6.44	0
1	1	1	152	17.082	9.17	0
1	1	1	1482	163.76	6.38	0
2	2	2	1037	117.338	6.92	0
1	1	1	339	37.816	4.97	0
1	1	1	1054	122.527	8.09	0
2	2	2	903	101.911	8.22	0
1	2	1	417	45.44	7.88	0
1	1	1	334	38.834	8.48	0
1	1	1	708	80.933	5.71	0
1	1	1	160	18.685	4.54	0
1	1	1	271	29.798	5.74	0
1	1	1	205	22.933	6.55	0
1	1	1	210	22.332	6.4	0
1	1	1	678	77.408	5.44	0
1	1	1	375	41.382	5.31	0
1	1	1	660	71.921	7.97	0
1	1	1	341	37.647	8.56	0
1	1	1	196	22.943	9.54	0
1	1	1	111	12.196	3.78	0
1	1	1	788	86.652	5.38	0
1	1	1	274	28.645	7.72	0
1	1	1	578	63.106	5.67	0
1	1	1	1494	166.692	6.79	0
1	1	1	1708	192.097	7.47	0
1	1	1	1047	116.329	6.54	0
1	1	1	1308	146.713	6.39	0
1	1	1	1129	124.267	6.61	0
1	1	1	828	89.036	6.81	0
1	1	1	1192	129.851	4.5	0
1	1	1	1705	191.771	6.04	0
1	1	1	803	89.955	7.09	0
1	1	1	351	40.71	4.97	0
1	1	1	1639	180.837	6.77	0
2	2	2	1025	115.657	8.27	0
1	1	1	1393	152.659	6.38	0
1	1	1	606	69.966	5.83	0
1	1	1	478	55.064	6.11	0
1	1	1	2752	299.438	12.06	0
1	1	1	494	56.645	11.52	0
1	1	1	238	27.35	11.82	0
1	1	1	1088	120.184	5.36	0
1	1	1	1503	164.799	4.48	0
1	1	1	445	51.731	8.9	0

1	1	1	302	34.528	8.25	0
1	1	1	881	96.664	4.55	0
1	1	1	289	31.985	8.68	0
1	1	1	354	39.356	5.6	0
1	1	1	429	48.604	9.58	0
1	1	1	432	47.455	6.99	0
1	1	1	851	97.855	5.49	0
1	1	1	418	46.145	7.43	0
1	1	1	522	59.033	5.15	0
1	2	1	387	45.274	5.11	0
1	1	1	1259	138.3	5.19	0
3	3	3	866	97.747	8.27	0
1	1	1	475	53.467	9.09	0
1	1	1	543	62.599	6.8	0
1	1	1	1026	117.328	5.55	0
2	2	2	1250	140.436	5.25	0
1	1	1	1735	185.586	7.75	0
1	1	1	1495	171.122	6.83	0
1	1	1	351	38.747	6.09	0
1	1	1	1701	189.985	7.21	0
1	1	1	1098	123.823	8.65	0
1	1	1	228	26.167	6.44	0
1	1	1	399	45.833	7.12	0
1	1	1	286	32.215	4.49	0
1	1	1	915	103.942	8.87	0
1	1	1	227	25.926	8.28	0
1	1	1	235	27.26	8.02	0
1	1	1	555	63.389	6.74	0
1	1	1	1124	125.019	7.18	0
1	1	1	528	59.106	7.05	0
1	1	1	494	56.033	5.3	0
1	1	1	1055	122.142	5.34	0
1	1	1	1066	122.482	5.24	0
1	1	1	200	22.393	5.44	0
1	1	1	165	17.876	7.44	0
1	1	1	1083	123.844	6.71	0
1	1	1	507	56.994	6.32	0
1	1	1	376	42.422	6.67	0
2	2	2	261	29.281	6.7	0
1	1	1	437	48.867	7.8	0
1	1	1	380	39.805	8.94	0
1	1	1	803	92.39	7.62	0
1	1	1	194	21.16	4.46	0
1	1	1	1173	136.316	7.44	0
1	1	1	943	106.032	6.64	0
1	1	1	677	74.843	5.57	0
1	1	1	585	63.822	9.04	0
1	1	1	810	89.076	8.37	0
1	1	1	707	78.529	4.87	0
1	1	1	1370	152.28	6.35	0
1	1	1	477	54.237	6.15	0
1	1	1	391	44.127	6.33	0

1	1	1	1837	197.173	6.8	0
1	1	1	342	35.373	5.6	0
